# Hybrid genetic algorithm-simulated annealing based electric vehicle charging station placement for optimizing distribution network resilience

**DOI:** 10.1038/s41598-024-58024-8

**Published:** 2024-04-01

**Authors:** Boya Anil Kumar, B. Jyothi, Arvind R. Singh, Mohit Bajaj, Rajkumar Singh Rathore, Milkias Berhanu Tuka

**Affiliations:** 1 Department of Electrical and Electronics Engineering, Koneru Lakshmaiah Education Foundation, Vijayawada, India; 2 Department of Electrical Engineering, School of Physics and Electronic Engineering, Hanjiang Normal University, Shiyan, 442000 Hubei People’s Republic of China; 3 Department of Electrical Engineering, Graphic Era (Deemed to Be University), Dehradun, 248002 India; 4 Hourani Center for Applied Scientific Research, Al-Ahliyya Amman University, Amman, Jordan; 5 Graphic Era Hill University, Dehradun, 248002 India; 6 Applied Science Research Center, Applied Science Private University, Amman, 11937 Jordan; 7 Cardiff School of Technologies, Cardiff Metropolitan University, Cardiff, CF5 2YB UK; 8 Department of Electrical and Computer Engineering, College of Engineering, Addis Ababa Science and Technology University, Addis Ababa, Ethiopia

**Keywords:** Electric vehicle, Charging station, Distribution generation, Photovoltaic, Genetic algorithm, Simulated annealing algorithm, Energy science and technology, Engineering, Mathematics and computing

## Abstract

Rapid placement of electric vehicle charging stations (EVCSs) is essential for the transportation industry in response to the growing electric vehicle (EV) fleet. The widespread usage of EVs is an essential strategy for reducing greenhouse gas emissions from traditional vehicles. The focus of this study is the challenge of smoothly integrating Plug-in EV Charging Stations (PEVCS) into distribution networks, especially when distributed photovoltaic (PV) systems are involved. A hybrid Genetic Algorithm and Simulated Annealing method (GA-SAA) are used in the research to strategically find the optimal locations for PEVCS in order to overcome this integration difficulty. This paper investigates PV system situations, presenting the problem as a multicriteria task with two primary objectives: reducing power losses and maintaining acceptable voltage levels. By optimizing the placement of EVCS and balancing their integration with distributed generation, this approach enhances the sustainability and reliability of distribution networks.

## Introduction

Due to their environmental benefits, energy-efficient operation, and amazing technological breakthroughs, EVs have recently become a major global phenomenon. EVs use stored electricity from rechargeable batteries instead of conventional ICE vehicles, which run on gasoline or diesel fuel to power their engines. This advancement in propulsion technology has significant effects on the environment, the energy sector, and the automotive industry^1,2^. EVs, which operate on the fundamental idea of harnessing electricity to propel an electric motor that drives the vehicle's wheels, represent a paradigm shift in the transportation industry. This power is kept in a large battery pack that can be recharged in a number of ways, like at home or in public outlets^3^. The vehicle can move because the motor effectively transforms electrical energy into mechanical power. There are many different types of EVs, such as BEVs, which run entirely on electricity without the use of an internal combustion engine; PHEVs, which combine electric and gasoline power for a variety of driving situations; and HEVs, which increase fuel efficiency through an electric motor but lack external charging capabilities^4^. Beyond being a technological marvel, EVs provide considerable environmental advantages because they have zero tailpipe emissions, which greatly reduce air pollution and greenhouse gas emissions. Additionally, because electricity is more affordable and they require less maintenance, they are more economically advantageous than traditional automobiles. Through developments in battery technology, EVs continue to increase their travel range in an effort to allay concerns about range anxiety^5^. While charging infrastructure continues to expand, customers are comforted by easy access to charging stations. With advancements in battery efficiency, quicker charging, and general performance, technological innovation in the EV sector is advancing at an unrelenting rate^6,7^. A new age in transportation is being brought in by the significant investments manufacturers are making in autonomous driving technology for EVs. Governments all across the world acknowledge the significance of EV adoption and provide incentives like tax credits, rebates, and access to travel-together lanes to promote their use. The continued advancement of battery technology ensures that EVs will become increasingly available and affordable to a wider consumer base, ultimately influencing the future of transportation as the automotive industry steers toward an electrified future, with automakers giving resources to electric models^8,9^. Unlike their onboard equivalents, offboard chargers are strategically placed outside the EVs and are typically found at specialized charging stations or charging infrastructure^10^. Their primary objective is to make it easier for high-power DC electricity to flow smoothly into the EV's battery. Offboard chargers effectively convert AC into DC before supplying this power, assuring compatibility with the vehicle's charging requirements^11^.

To address the pressing challenge of integrating renewable energy sources with electric vehicle charging stations, our study introduces a novel hybrid Genetic Algorithm and Simulated Annealing approach (GA-SAA). Unlike existing methods, our approach optimizes the placement of EVCS in distribution networks by significantly enhancing the system's resilience and efficiency. This study uniquely contributes to the existing body of knowledge by providing a comprehensive comparison with traditional methods, thereby highlighting our methodology's superior performance in reducing power losses and maintaining optimal voltage levels.

Offboard chargers excel in terms of charging speed thanks to their capacity to offer quick charging options^12^. It evolved especially for Level 3 charging, also referred to as DC fast charging. With this degree of charging ability, EVs may charge extremely quickly, sometimes in less than 30 min, and can frequently charge up to 80% of their batteries^13,14^. Offboard chargers are a vital part of the EV charging ecosystem because of their high-speed charging capabilities, which meet the needs of drivers looking for convenient and speedy recharging choices^15^. When it comes to technology, it is usual and practicable to employ an AC bus with numerous AC/DC conversion stages, especially when working with electricity sourced from the common AC power grids. The efficiency of the system as a whole might be impacted by the energy losses introduced at each conversion stage; it is crucial to note^16,17^. The benefits of using AC technology must be carefully weighed against the need to reduce these losses for optimum performance by engineers and designers. Economically, choosing an AC bus with numerous conversion steps can greatly impact how much money is spent on infrastructure^18^. Although AC technology is well-established and standardized, adding several conversion stages raises upfront costs due to complexity and the requirement for additional parts. The early expenditures of implementing established technological standards must be compared to the long-term advantages for organizations^19^.

**Figure 1:**
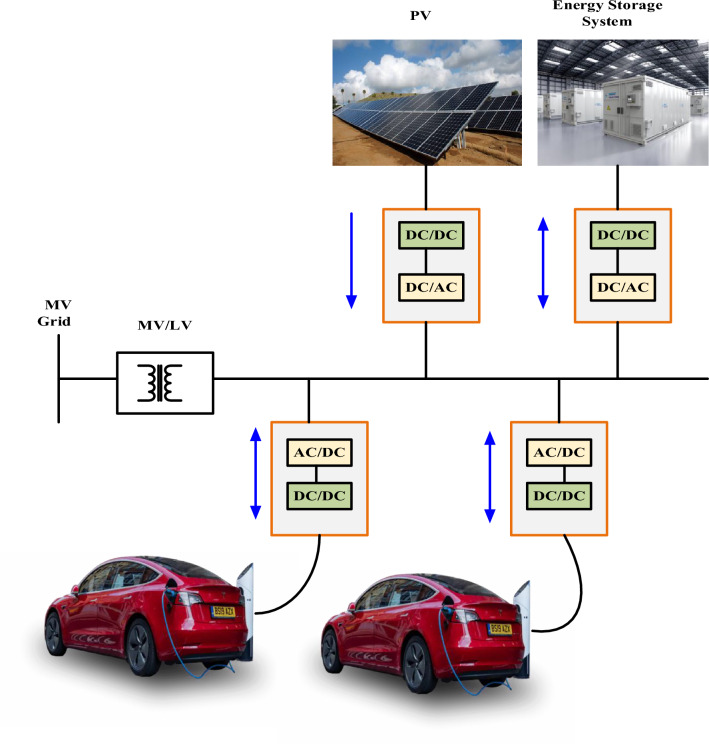
Ac bus distribution system.

Efficiency is crucial, especially in applications that relate to energy. Higher energy losses from more conversion stages typically result in higher energy consumption and operational costs^20,21^. Minimizing conversion stages becomes a focus to improve total system efficiency and lessen environmental effects in applications where efficiency is critical, such as renewable energy generation. The availability of AC switchgear and safety equipment directly impacts the dependability and safety of an electrical system^22,23^. The system must be protected against defects, and staff safety must be guaranteed using standardized methods and devices. These elements help the electrical distribution system to be stable and reliable overall, essential for consistent functioning and public safety^24^. An increased system's energy losses can have detrimental effects on the environment. Particularly when electricity is produced from non-renewable resources, increased energy use raises costs and increases carbon footprint. In applications where sustainability and reduced environmental effect are crucial considerations, minimizing conversion losses can be seen as a responsible environmental option, aligned with the overarching objective of developing a greener and more eco-friendly energy landscape, as shown in Fig. 1. DC-connected systems make a strong technical argument thanks to their streamlined electrical routes and minimized conversion stages, which result in improved efficiency^25,26^. This inherent efficiency is very useful when it comes to energy storage technologies like batteries, which are designed to run on DC power^27,28^.

**Figure 2:**
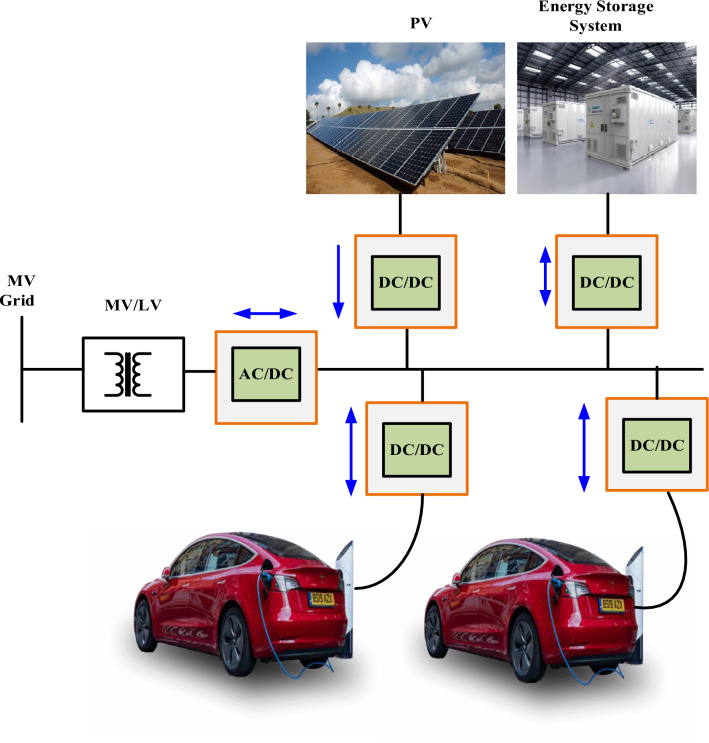
DC bus distribution system.

Energy losses are reduced, and the simplified architecture of DC systems improves overall performance^29^. Systems using DC connections provide significant long-term cost savings in the field of economics. Due to the central front-end converter, initial installation costs might be similar to or slightly higher, but as a result of reduced energy losses and simplified componentry, operational costs may be lower, and the return on investment may be higher^30,31^. Consider the extended durability and stability of DC infrastructure in long-term energy projects, and this savings becomes even more apparent. Last but not least, DC systems promote innovation, enabling breakthroughs in fields like grid integration strategies and fast-charging technology^32,33^. As a result of their versatility, they are a flexible and progressive option for contemporary energy infrastructure since they can keep up with changing trends and specifications in the rapidly changing electrical industry, as shown in Fig. 2^34^.

A variety of problems and unfavorable outcomes may result from the improper placement of EV charging stations inside the distribution network. First of all, a disproportionately high concentration of charging stations in a few locations can overwhelm the current distribution infrastructure, resulting in Grid Overload. Voltage changes, equipment problems, and interruptions in service could all come from this. Another issue is voltage variations. Voltage instability in the distribution network might result from an uneven deployment of charging stations. The quality of the electricity sent to users, the possible injury to delicate electrical equipment, and the operation of appliances can all be negatively impacted by groups of charging stations demanding a lot of power. The power draw of charging stations, especially fast-charging ones, can put a lot of strain on nearby substations and transformers. These stations can overload surrounding transformers and substations if they are built without considering capacity constraints. This could result in equipment failures and overheating, which would require expensive repairs and downtime. The uneven deployment of charging stations may cause an unbalanced load over the distribution network. While some feeders might be fully utilized, others might be lightly laden. Inefficiencies may occur from this mismatch, and fixing it might involve expensive infrastructure upgrades. Another issue is ineffective resource use. Poor resource allocation, greater operating costs, and eventually higher costs for customers can arise from suboptimal placement, which can leave charging stations underutilized in certain places and overloaded in others.

The impact of plug-in behaviors on future electric vehicle (EV) charging load profiles and spatial–temporal flexibility is examined in this study. The research considers technological diffusion stages and geographical situations in a variety of settings using an agent-based model. The 'SOC' scenario exhibits the highest potential, and the results show that plug-in behaviors considerably impact peak reduction and load shifting. According to the study, driving behavior has a bigger influence on EV charging loads than plug-in behavior, underscoring the significance of taking it into account. It is advised that more study be done on incentives, controlled charging, and mobility patterns in order to fill in any gaps in the present analysis^35^. Furthermore, utilities may suffer a major financial hit from the placement of EVCS, which is less than ideal. It frequently calls for substantial investments to modernize the infrastructure, like adding more substations, transformers, and distribution lines. Consumers familiar with the difficulties of switching to EVs may see an increase in their bills due to these essential expenses pushing up utility rates. In addition to the financial strain, the appearance of unfair billing is a worrying result of poor placement. This means that although certain areas may have easy access to charging infrastructure, others may only have limited or no access, making EV adoption convenient and desirable. This disparity in charging accessibility impedes not only the widespread adoption of EVs but also the effort being made by everyone to cut emissions and rely less on fossil fuels. A fair and balanced deployment of charging stations is essential to guarantee that everyone, regardless of location, can take advantage of EV technology. The risk of high power losses and voltage fluctuations, problems that can occur when EVs are charged without a well-thought-out plan, can be reduced to a minimum by strategically integrating EVCS within distribution networks. Research in the past has mainly embraced heuristic strategies while looking for a solution. These methods provide useful and understandable answers, making them especially helpful for quickly addressing pressing short-term scheduling and planning issues pertaining to EV charging infrastructure. In this situation, heuristic algorithms play a crucial part in managing the impact of EVCS. In light of the rising popularity of EVs, it is vital to maintain the dependability and stability of distribution networks.

When it comes to power losses and voltage swings, haphazard EV charging practices can pose serious problems for the distribution network. Concurrently charging several EVs can put a strain on the system, increasing resistive losses in conductors due to a larger current demand, especially if it is done without coordination. Furthermore, when many EVs are linked, energy conversion losses from AC/DC converters, which are crucial elements of EV charging stations, accumulate and reduce overall efficiency. Brownouts or problems with consumer electronics can happen from voltage decreases that exceed allowable limits as a result of power outages. On the other hand, aggregating EVs in locations with low electrical demand might raise voltage levels, increasing the danger of appliance damage and raising safety issues. Sensitive devices can be harmed by rapid voltage fluctuations caused by EVs that are disconnected or charged abruptly. Adherence to regulatory standards is essential to reducing these problems, guaranteeing that power losses and voltage variations stay within permitted limits and preserving the stability and dependability of the distribution network as EV usage rises^36^. In order to address issues in the power sector, strategically placing EVCS has become increasingly important as the adoption of EVs has increased globally. In order to optimize CS placements, this work offers a unique method that uses the TLBO algorithm and focuses on minimizing power loss, lowering voltage deviation, and boosting voltage stability for a sustainable energy ecosystem. In simulations on IEEE 33-bus and 69-bus systems, TLBO regularly outperforms other heuristic algorithms, demonstrating the importance of efficient CS allocation. In the future, research will focus on integrating EVs' most efficient and environmentally friendly charging features^37^. In order to maximize PEV integration into distribution networks, this study introduces the DIEM strategy. For both nodal aggregators and distribution system operators, DIEM uses two optimization layers with an emphasis on actual and reactive power management. By utilizing the reactive power capacity of PEVs, DIEM improves voltage management, reduces power loss, and allows for higher PEV penetration while staying within voltage constraints. In comparison to the conventional SIEM approach, DIEM provides system operators and PEV owners with improved performance advantages while preserving battery life. This advancement is expected to revolutionize PEV integration into distribution systems, improving sustainability and efficiency^38^. This study underlines how vital it is to design effectively for PEVCS, in particular FCSs, in an era of increased interest in EVs. Nash bargaining theory is used to examine the DISCOs and FCS owners, utilizing a sophisticated approach that models the FCS planning challenge as a mixed-integer nonlinear programming problem. This approach generates fair profits for both parties by optimizing location, size, and energy pricing. It also proposes potential incentives for greater benefits. The potential for wider application to multiple PEV charging station types in the changing EV infrastructure landscape provides an invaluable tool for DISCOs, FCSOs, and decision-makers involved in FCS planning^39^. The placement and sizing of charging stations need to be based on thorough scientific investigation in order to achieve widespread adoption of EVs. This study presents a novel method for planning rapid charging stations that considers the operator's economics, the driver's satisfaction, the vehicle's power consumption, traffic management, and grid stability. An important distinction is that it uses dynamic, real-time data rather than static statistics, giving planning a stronger grounding. A global simulation platform is used to assure applicability in different locales. A real-world case study in Beijing reveals how this strategy offers a complete answer to the planning of EV charging infrastructure by optimizing operator revenues, boosting driver satisfaction, and enhancing traffic efficiency and grid safety^40^. Due to India's population growth and environmental concerns, strong charging infrastructure is required as the market for EV’sexpands. With a multi-objective framework that takes into account economic issues, grid stability, dependability, and EV user convenience, including traffic circumstances, this study focuses on Guwahati, a developing smart city. The impact on the power grid is reduced while EV driver convenience is prioritized using a hybrid algorithm that combines CSO and TLBO. Future initiatives will focus on price plans, V2G charging, and renewable energy-powered stations to promote EV adoption further in Guwahati and other Indian cities^41^. FCSs must be strategically placed due to the rise in EVs in order to prevent grid issues. The challenging challenge of jointly identifying and scaling DGs and FCSs while accounting for EV numbers and network constraints is undertaken in this study. It improves voltage profile upgrades, network power loss, FCS costs, and EV user happiness using the NSGA-II. A win–win scenario for owners of EVs and charging stations is created by combining FCS and DG deployment, which reduces grid stress from heavy EV use while boosting grid stability^42^. In order to solve issues with accessibility and grid stability, this study focuses on the best locations for EV charging stations. To produce Pareto optimal solutions for cost reduction, dependability, and power loss, as evaluated by the VRP index, a hybrid CSO TLBO algorithm is used. The choice of solutions is improved through fuzzy decision-making. This study not only presents a multi-objective framework but also illustrates the efficiency of the hybrid algorithm, making it a useful tool for encouraging EV adoption through strategically positioned charging infrastructure. Future work will broaden its application to other placement challenges to demonstrate its adaptability and effectiveness in handling challenging optimization tasks^43,44^. In light of the increased usage of EVs, this article examines the stability of the distribution network. In order to improve voltage profiles, reduce power losses, and reduce costs, it suggests a novel positioning technique for solar-powered charging stations. It works well on a 33-bus system, cutting down on power losses and enhancing voltage profiles by integrating probabilistic modeling for EV load prediction with a feed-forward neural network for solar power estimation. In-depth models that consider EV driver behavior and hybrid algorithms for urban charging station placement may be explored in future studies. Comparative analyses have shown that the modified CSO method is preferable^45^. Smart cities have a unique potential to change with the widespread use of EVs, but effective charging infrastructure deployment is essential^46^. Rapid charging is provided via fast chargers (level 3). However, system parameters may be strained by their random location. The novel strategy presented in this research manages EV loads effectively and minimizes costs, losses, and transformer congestion while optimizing all charger types. Using PSO, it integrates PV generation while using stochastic modelling to account for user uncertainties in the EV load. The results demonstrate significant cost savings, decreased daily losses, and reduced transformer congestion using real-world data from NUST Pakistan's distribution system. By delaying the need for new transformers and employing distributed PV power to improve EV charging infrastructure while maintaining grid stability, this adaptive solution helps both business and residential environments^47^. In order to strategically deploy EV charging stations in distribution networks with randomly distributed rooftop solar panels, this study suggests the hybrid optimization algorithm BFOA-PSO. The study results show the algorithm's efficiency in reducing power losses and preserving voltage stability, which helps ensure that EVs are easily integrated into contemporary distribution networks^48^.

The smooth integration of Electric Vehicle Charging Stations into modern distribution networks is a complicated task that necessitates strategic planning. With the growing popularity of electric cars (EVs), the necessity for well-coordinated EV infrastructure expansion has become important. This expansion is critical for the widespread acceptance of EVs and addressing the pressing environmental challenge of reducing greenhouse gas emissions related to conventional petroleum-based vehicles. The assessment of the literature emphasizes the critical need for an effective and strategically planned growth of EV infrastructure, recognizing the delicate interplay between distribution networks and EVCS integration. Understanding and tackling the issues of this integration are becoming increasingly important as worldwide EV adoption accelerates. The problem formulation, which is based on literature insights, revolves around optimizing the placement of plug-in EV charging stations within distribution networks. This optimization is critical for mitigating the potential negative impacts of EVCS integration on network stability, power losses, and voltage deviations. The presence of dispersed Photovoltaic systems complicates the strategic placement of EVCSs even further. Since there are multiple objectives involved in this problem, it is necessary to reduce both active and reactive power losses at the same time while making sure voltage levels stay within reasonable bounds. In order to improve the overall efficiency and reliability of distribution networks, the research aims to achieve a careful balance between the demands of EVCS integration and the integration of dispersed generation. A detailed examination of the litertature review of Optimizing Electric Vehicle Charging Stations has presented in Table 1.

**Table 1:** Research studies optimizing electric vehicle charging stations.

References	Focus	Methodology	Key findings	Research implications
^37^	Placement of the CS, power losses, and voltage swings	TLBO algorithm	Optimizing CS placements	It explore integrating efficient , environmentally friendly charging features
^43^	EV charging station locations, grid reliability	Fuzzy decision-making with the hybrid CSO TLBO algorithm	Pareto ideal solutions for power loss, cost reduction	
^45^	Distribution network stability and placement of solar-powered charging stations	Probabilistic modeling using feed-forward neural networks, modified CSO approach, and EV load prediction	lower power losses and better voltage profiles on a 33-bus system	Using hybrid algorithms to locate charging facilities in cities and in-depth models to analyze the behavior of EV drivers
^47^	Grid stability, cost savings, and efficient implementation of charging infrastructure	Stochastic modeling and PV generation integration with PSO	Significant cost reductions, a reduction in transformer congestion	adaptable solution that delays the need for new transformers in both residential and commercial settings
^48^	EV charging stations with solar panels strategically placed	Hybrid BFOA-PSO optimization algorithm	Effectiveness in maintaining voltage stability and reducing power losses	Make it easier for EVs to be integrated into modern distribution networks

The optimization of EVCS placement for several objectives, including reducing power losses or voltage fluctuations, was the main focus of studies like^37^ and^43^. It is possible that the integration of solar power generation with the charging stations is not specifically taken into account in these studies. In^45^ focus mainly on network stability when solar-powered charging stations are integrated and Maybe not a full optimization with the goal of maximizing sustainability. In^47^ optimizes grid stability and cost savings by combining PSO with stochastic modeling, a technique that takes random elements into account. To optimize the placement of EV charging stations with solar panels,^48^ uses a hybrid BFOA-PSO method, which combines PSO and Bacterial Foraging Optimization. Although renewable energy is mentioned in the literature, it doesn't go into great detail about how solar power and EVCS work together. This gap offers a chance to improve the infrastructure for EV charging's sustainability. Research on EVCS location optimization is severely lacking because IEEE network dynamics and features are often ignored. These factors would make it possible to locate charging infrastructure more precisely and effectively.

Based on the above research gaps, the novely of this reserach is as follows,

- By concentrating on the complementary effects of solar power and EVCS installation, this study goes further.
- In order to gain benefits, it is necessary to investigate how these two factors might be integrated and optimized.
- One possible way to reduce EV charging's reliance on the main electrical grid is to locate charging stations in close proximity to solar power sources.
- It focuses on optimizing solar power integration and EVCS placement in conjunction to maximize sustainability and network stability tries to increase the system's overall sustainability through careful placement and optimization of the charging infrastructure.
- Hybrid optimization algorithms are used in references^47,48^, and proposed research. The paper proposes a novel hybrid GA-SAA approach to the complex optimization task. This hybrid technique effectively navigates the complicated solution space by leveraging the strengths of both algorithms. The choice of GA-SAA in the proposed study will support its usefulness to multi-objective optimization techniques like
  - Active and reactive power losses
  - Voltage deviation index
  - Optimal placement of charging stations and PV system.
- *Suitability of GA-SAA*
- GA's strengths
  - Exploration
    Within the search space, the genetic algorithm component is particularly good at examining a large number of potential solutions. Because the ideal location might not be apparent right away, this is quite important.
  - SAA's strengths
  - Exploitation

By concentrating on the potential regions that the GA found, simulated annealing helps in search refinement. For optimal results, it enables "fine-tuning" the locations of stations and PV system. Combining GA and SAA results in GA-SAA, an effective method. While SAA focuses its search inside those areas to identify the optimal configuration, GA searches the large search space for promising areas. When minimizing multiple objectives at once, GA-SAA can handle it well and develop a solution that balances all the desired outcomes. The comparison of GA-SAA alogrithms with PSO and BFOA-PSO detail Although PSO works well, it may have trouble escaping local minima and dealing with complicated search spaces. In this case, SAA's capacity to escape local minima and GA's exploration are advantageous. BFOA may encounter difficulties with complex search spaces, much like PSO. GA has an advantage due of its exploration capabilities. The study tackles the problem of EVCS integration in distribution networks with distributed PV systems. The suggested method optimizes the strategic positioning of EVCSs while maintaining the efficiency of PV systems, ensuring harmonious coexistence. This study offers a novel hybrid optimization algorithm that combines the GA-SAA**.** The major goal of this study is to identify the best locations for distribution network-based EVCSs. In the presence of rooftop PV systems that are randomly sized and distributed, these EVCS deployments are tactically optimized. The case study network is the feeder of the IEEE 33 test network. The research contributes to understanding the delicate balance required between the integration of EVCSs and the stability of distribution networks. This is achieved by strategically placing PEVCS to minimize power losses and voltage deviations. The paper presents results and discussions based on real-world scenarios after conducting a thorough simulation using MATLAB 2018a. The validation of the proposed GA-SAA approach against other methods improves the findings' reliability and applicability.

In light of recent advancements, our review identifies a gap in the application of hybrid optimization algorithms for EVCS placement within power distribution networks incorporating renewable energy sources. Notably, few studies have simultaneously explored the integration of Genetic Algorithms and Simulated Annealing techniques to achieve a balance between exploration and exploitation in search spaces. This paper aims to bridge this gap by demonstrating the efficacy of a hybrid GA-SAA method in optimizing network resilience and renewable energy integration.

The structure of this paper is as follows: The Methodology is covered in detail in Section "Methodology", the Allocation of PEVCS is covered in Section "Allocation of PEVCS", the Optimization Methodologies are covered in detail in Section "Optimization methodologies", the Hybrid Algorithm is explained in detail in Section "Hybrid GA—SA algorithm", and the Results and Discussions are covered in Section "Results and discussions".

## Methodology

### Networks under investigation

Integrating PEVCS into RDNS is a complex task with a number of important factors to take into account. The distribution of load and variability within the RDNS is first made more complex by the addition of PEVCS. The timing and the number of vehicles connecting to the grid can show large changes, even if the charging patterns of EVs are generally predictable. Preserving grid stability in the face of such fluctuating demand calls for more reliable load forecasting. Furthermore, the concentration of many EVs charging at once can cause peak demand surges that, if properly managed, might put a burden on the current RDNS infrastructure. Upgrading transformers, distribution lines, and substations may become necessary to handle the increasing load without jeopardizing system dependability. Voltage regulation is yet another essential RDNS component that PEVCS impacts. Voltage fluctuations caused by excessive charging at a specific location must be controlled within allowable bounds to prevent equipment damage and provide a steady supply of high-quality electricity to customers. A further level of complication is added to RDNS administration by some EVs' ability to provide bidirectional power flow. While this technology allows grid services like peak shaving and grid stability, it also necessitates complex control systems to manage power flows successfully. For RDNS operations to be optimized and potential problems to be minimized, PEVCS placement must be strategic. Effective load distribution can be achieved by locating areas with sufficient capacity and few grid restrictions. Furthermore, implementing intelligent charging algorithms and demand response programs can reduce the negative effects of PEVCS on the RDNS. In order to regulate charging schedules and encourage off-peak charging, grid operators may now do so using these technologies, which ease the load on the grid during peak usage. Expanding PEVCS infrastructure requires careful consideration of both the costs and advantages. To ensure that costs and benefits are divided equitably and promote sustainable growth in the EV sector, the best tariff structure must be chosen for PEVCS operators and EV owners.

The additional load from PEVCS also coincides with objectives for environmental sustainability, notwithstanding the difficulties it creates. In comparison to conventional internal combustion engine vehicles, EVs are frequently regarded as a cleaner alternative, with the potential to cut greenhouse gas emissions and promote a more sustainable transportation system. To ensure the successful integration of PEVCS into the power distribution landscape as RDNS develops, it will be essential to address these many-faceted issues.

### IEEE 33 bus network

As defined by the IEEE, "Standard IEEE networks" are power distribution networks that have been methodically planned and strictly governed. As a well-known professional organization, the IEEE is particularly interested in the power and energy industry, but it also plays a critical role in creating and distributing standards across a wide range of businesses. These standards act as crucial benchmarks to ensure the safe, dependable, and effective operation of electrical systems and consequently help maintain the overall stability of the power grid. The "IEEE33" network possesses a special position inside this architecture^49^. In the field of studying and researching power systems, it serves as a frequently used test distribution network. A typical real-world distribution network has 33 nodes, which can be compared to substations or busbars. This network is a simplified representation of that network. Each of these nodes represents a key location within the distribution system where electrical power is either produced, consumed, or distributed, and power lines connect them. The IEEE33 network's goal is to make experimentation and analysis easier by providing researchers with a controllable yet realistic model to study and improve the operational features of distribution networks. For researchers sorting through the complex realm of RDNS, the IEEE33 network provides an invaluable laboratory and standardized framework. Its radial or tree-like form closely resembles the topological make-up of RDNS in the real world, providing a concrete model to understand how these networks function in a variety of situations. The IEEE33 network serves crucial functions in numerous aspects of RDNS analysis outside of its structural representation. In order to investigate how various load kinds, from residential to industrial, interact with RDNS, it allows for realistic load modeling and offers a common interface. Researchers can carefully analyze voltage profiles under various conditions thanks to the monitoring of voltage control, a key component of a steady power supply. The inclusion of dispersed generation nodes aids in evaluating the effect of renewable sources on grid stability, and fault analysis aids in the implementation of effective preventive solutions. In addition to serving as a testbed for smart grid technologies, feeder automation methods are optimized for quick problem detection. This opens the door for cutting-edge sensors, communication networks, and control algorithms. The IEEE33 network is still a crucial resource for comprehending and planning the future of electrical distribution networks as RDNS adapts to incorporate contemporary technologies. The entire power of each network is shown in Table 2.

**Table 2:** IEEE 33 bus total power demand.

Bus type	Active power (kW)	Reactive power (k VAR)	Apparent power (KVA)
IEEE33bus	3715	2300	4369.35

The IEEE 33-node test distribution network works at a voltage of 12.66 kV while preserving balance" indicates that this particular distribution network follows the foundations of a balanced electrical system. Both equality in the magnitudes of voltages and currents across all phases and a symmetrical phase arrangement with evenly spaced phase angles at 120-degree intervals are present in a balanced system. This equilibrium is a crucial part of effective and trustworthy power distribution since it efficiently addresses issues like variations in voltage and ensures reliable power transfer. The IEEE 33-node test distribution network is made up of 33 interconnected nodes or buses and is well-known in the field of power system analysis. As a common framework for evaluating and testing various methods, models, and methodologies in the field of power distribution analysis, this network is frequently used by researchers and engineers. This distribution network's operational voltage, which falls solidly within the medium-voltage spectrum, is indicated by the stipulated voltage rating of 12.66 kV. Comprehensive electrical testing and measurements are typically performed to support the claim of equilibrium within this network. These assessments include measuring the voltages and currents at various system junctions to confirm that they satisfy the requirements of a balanced system, including equality in magnitude and symmetrical phase angles throughout the three phases^50^.

**Figure 3:**
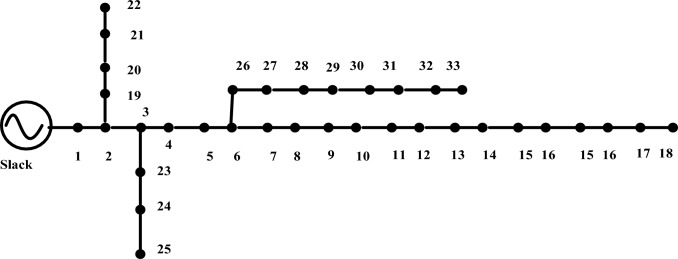
IEEE 33 node test feeder.

In essence, the IEEE 33-node test distribution network's equilibrium is maintained at 12.66 kV, which is essential for maintaining its dependability and effective operation, as shown in Fig. 3. This equilibrium successfully mitigates unwanted events like voltage mismatches and uneven power distribution. As a result, to better understand distribution networks' behavior and develop plans to improve their overall performance and stability, researchers and professionals from the industry rely on balanced test systems like these.

A crucial measure that reveals the proportion of the distribution network's total load that can be assigned to residential customers is the residential load percentage. This statistic is extensively relied upon by network planners and operators as it helps to carefully manage energy needs across residential, commercial, and industrial sectors, ultimately helping to fine-tune resource allocation techniques and enhance grid stability. It's crucial to remember that the specific percentage can vary significantly depending on the degree of urbanization, the area, the time of day, and the dominant local economic activities. The complex geography of electrical consumption inside the network is shaped by these dynamic components taken as a whole. Residential loads are important since it account for 85% of all network loads that serve residential and commercial purposes.

With the residential loads accounting for 85% of the total power in IEEE 33 network is calculated as follows,

- Active power (kW): 3715 * 0.85 = 3157.75
- Reactive power (kVAR): 2300 * 0.85 = 1955
- Apparent power (KVA): 4369.35 * 0.85 = 3713.9

The above total power demand of each network's residential loads is shown in Table 3^45^.

**Table 3:** Residential power demand.

Bus type	Active power (kW)	Reactive power (k VAR)	Apparent power (KVA)
IEEE33bus	3157.75	1955	3713.9

### Estimation of the number of PEVs and the required number of charging points:

Understanding a community's power needs is important in many circumstances, particularly in urban planning, electrical engineering, or energy management^51^. Making energy-efficient decisions and planning electrical infrastructure are made easier with the use of this knowledge. Based on a community's power consumption, the number of homes (Nh) is calculated using the formula presented in Eq. (1).

1 $$N{\text{h}} = \frac{S{\text{th}}}{S{\text{h}}}$$

where N_h_ is the number of households in the study area.S_Th_ is the total power demand of residential loads.S_h_ is the power demand of a single household.

Here are some statistics on how many electric cars (EVs) are estimated to be present in a neighbourhood with a 59% integration rate calculated in Eq. (2).

2 $$\%EV = \frac{N{\text{hEV}}}{N{\text{h}}}$$

For the sake of simplicity, only 100 models were chosen for evaluation out of the 184 EVs that might be produced from Eq. (2). Five real-time EV models were chosen for the assessment's thorough evaluation, as listed in Table 4^52^.

**Table 4:** Some EVs and their charging characteristics.

S. No	EV model	EV specifications	Charging type—level 2 (22 kW)		kW
			Charging time	Quantity	Required kW for EV model
1	Mg Comet EV	17.3 kWh, 230 km	7 h	20	2.47
2	Citrogen EV	29.2 kWh, 320 km	10h 30min	20	2.76
3	Tata Tiago EV	19.2 kWh, 250 km	6.9 h	20	2.78
4	Tata Nexon EV	30 kWh, 312 km	10.5h	20	2.8751
5	Hyundai IONIQ	72.6 kWh, 631km	6 h 55min	20	10.49
				Total = 100	Total kW = 21.3751

### EV’s characteristics

Regarding the infrastructure for EV charging, a total of 5 EVCSs are strategically positioned throughout the network to accommodate 100 EVs. Although they may have a different number of CPs, these charging stations use Level 2 chargers.

*Step 1* Number Of Charging Ports Per EVCS

Each EV (Electric car) charging station has a certain number of charging ports, as given in Eq. (3), or locations where an electric car may plug in and recharge its batteries.

A simple calculation has been defined here based on individual kW in Table 4.

3 $$\begin{aligned} {\text{Number~of~charging~ports~Per~EVCS}} = & {\text{~}} & \frac{{{\text{Individual~Quantities~wrt~to~particular~kW}}}}{{{\text{Total~power~needed~for~level~}}2{\text{~kW}}}} \\ = ~\frac{{\left( {20*10.49} \right)~ + ~\left( {20*2.47} \right)~ + ~\left( {20*2.78} \right)~ + ~\left( {20*2.78} \right) + ~\left( {20~*~2.8751} \right)}}{{22}}~ = ~20 \\ \end{aligned}$$

Number Of Charging Ports Per EVCS is ‘20’.

*Step 2* Rating Of EVCS

The electrical capacity or power output of an EVCS is often referred to as its "rating". It's a crucial parameter that shows how quickly an EV’s charging station can deliver electricity, which is given in Eq. (4). Typically, this rating is expressed in kW.

4 $$\begin{gathered} {\text{Rating~}}\;{\text{of}}\;{\text{EVCS~}} = {\text{~Rating~}}\;{\text{of~}}\;{\text{CP}}\;{\text{~for}}\;{\text{~level~}}2\;{\text{~*~}}\;{\text{Number~}}\;{\text{of~}}\;\;{\text{Charging}}\;{\text{~Ports~}}\;{\text{Per~}}\;{\text{EVCS}} \hfill \\ {\text{Rating~}}\;{\text{of~}}\;{\text{EVCS~}} = {\text{~}}22{\text{~*~}}20{\text{~}} = {\text{~}}440\;{\text{~kW}} \hfill \\ \end{gathered}$$

*Step 3* Number Of EVCS

The "number of EV charging stations" refers to the total number of individual stations or sites where EVs can be charged. Owners of EVs can plug in their vehicles and charge their batteries at these stations because they are set up with charging infrastructure given in Eq. (5).

5 $$\mathrm{Number\; of\; EVCS}= \frac{\mathrm{Total\; Number\; of\; EV{\prime}}{\text{s}}}{\mathrm{Number\; of charging\; ports\;per\; EVCS}} = \frac{100}{20} = 5$$

*Step 4* Total Rating

The total electrical capacity of all EVCS within a specific area or network is known as the "total rating of EVCS" and is frequently expressed in kW or MW. It denotes the maximum power output for recharging EVs that all of the charging stations in that area or network are capable of providing individually as given in Eq. (6).

6 $$\mathrm{Total\; Rating }\left({\text{kW}}\right)=\mathrm{ Rating\; of \;EVCS }*\mathrm{ Number\; of\; EVCS }= 440*5 = 2200\;\mathrm{ kW}$$

Based on the above calculation, all parameters with numerical values listed in Table 5.

**Table 5:** Distribution of level 2 charger charging types.

Charger	Rating of CP (kW)	Number of CP per EVCS	Rating of EVCS (kW)	Number of EVCS	Total rating (kW)
Level 2	22	20	440	5	2200

### Penetration of PV systems, along with randomized sizing and placement

A cutting-edge method of optimizing renewable energy systems, seen from a broader viewpoint, is the use of MATLAB to model PV systems in negative PQ modes with a power factor of 0.95. This technical decision highlights the significance of accuracy and cutting-edge equipment in attaining grid stability while maximizing renewable energy production.

The energy industry's significant shift towards sustainability is exemplified by the penetration of PV systems reaching 60% on the grid. The considerable amount of renewable energy sources necessitates a fundamental revaluation of grid management and operation, with grid operators adjusting to meet the variable nature of solar power generation. This transition depicts how our energy system is always changing to move toward a more reliable and kind future to the environment^53^.

- *Total Production of the PV Systems to Total Generation* The ratio of the electricity produced by PV systems to the total amount of electricity produced in a given area or system is known as the total production of the PV systems to total generation. It aids in quantifying solar energy's contribution to the overall energy supply.
- *Peak PV Capacity to the Load's Peak Apparent Power* This shows how the electrical load's highest apparent power consumption compares to the PV system's maximum capacity or the amount of power it can generate. It's a method of determining whether the PV system can supply the linked devices' peak power requirements.
- *PV-rated power to the load's active power demand* This gauges the PV system's capacity to satisfy the load's active power (real power) requirements. It informs users if the solar system can produce enough energy to meet the needs of the electrical devices.

The below calculations show that in addition to providing 771.19 kVAR is given in Eq. (8) of reactive power to the grid, PV systems with a power factor of 0.95 and a rating of $2346.315$ kW is given in Eq. (7), which account for 60% of the electrical demand, also satisfy this need. The fact that they also enhance grid stability and energy provision emphasizes their dual role in this regard.

7 $$\mathrm{Total\; Rated\; power}({\text{kW}})= \frac{\mathrm{pv \;penetration }}{\mathrm{power\; factor }} *\mathrm{ Active\; power \;demand }= \frac{0.6}{0.95}*3715 =2346.315\mathrm{ kW}$$

8 $$\mathrm{Total\; Reactive\; power }\left({\text{kVAR}}\right)=\mathrm{ kW }*\mathrm{ tan}\left({\text{angle}}\right) = 2346.315*0.328=771.19\;\mathrm{ kVAR}$$

Randomly placed rooftop PV systems are emphasized as a crucial component in the research. Only nodes with electrical loads are given these PV systems in the study network; nodes without loads are not considered. In order to improve energy distribution and efficiency, each PV system is built with 330W PV modules, allowing for variable allocation and positioning of these systems throughout the network. It is noteworthy how this study makes use of the *rand()* function. In order to provide a dynamic allocation of 330W PV modules to certain target nodes, it is used to incorporate a random element. In order to maintain a total network PV power rating of 2346.315 kW, Eq. (9) is then used, demonstrating a versatile and adaptive strategy for maximizing solar energy distribution across the network.

9 $$n{\text{a}} = \frac{rand(a)}{\sum_{a=1}^{33}rand(a)}*k$$

This framework denotes by "n" the number of PV modules assigned to a specific node "a." Individual nodes "a" are given random values between 0 and 1, which are produced by the function "rand(a)," and are then given those values. To guarantee that the total PV capacity across the network precisely equals 2346.315 kW, the parameter "k" is a carefully chosen constant.

The total distributed PV capacity, P_T_ as well as the PV capacity at each node, P_pva_ are defined in Eqs. (10) and (11)

10 $$P\text{pva }= na * P{\text{pvm}}$$

PPVa stands for the PV capacity at node "a," where "a" is the quantity of PV modules on that particular node, and PPVm represents the 330W rating of a single PV module.

11 $$P{\text{T}} = \sum_{a=1}^{33}P{\text{pva}}$$

## Allocation of PEVCS

### Problem formulation

A well-defined optimization issue must first be created, and it must then be solved using mathematical optimization techniques in order to optimize a power system to lower active and reactive power losses while also reducing voltage variations. Let's examine this procedure in greater detail by thoroughly breaking down the issue and all of its component parts. The objective function, which expresses the main objective—minimization—is at the center of this optimization effort. In this case, a combination of crucial elements must be minimized.

(A) Objective function (Minimization)
(B) Active power loss

These losses result from energy loss caused by the innate resistance of transmission and distribution cables. They can be expressed as the total active power losses that occur in all network branches, and they have a direct relationship to the square of the current flowing through the lines.

12 $$P{\text{loss(i)}} = i_{{\text{i}}}^{2} *{\text{R}}_{{\text{i}}}$$

(ii) Reactive power loss

These are caused by the movement of reactive power within the system instead of active power losses. They can be analytically stated in a manner comparable to active power losses and are connected to the square of the reactive power.

13 $$Q{\text{loss(i)}} = i_{{\text{i}}}^{2} *{\text{X}}_{{\text{i}}}$$

The cumulative active and reactive power losses are denoted by P_Loss(i)_ and Q_Loss(i)_, respectively, while Ri and Xi represent the ith branch's resistance and reactance. 'i' stands for the branch number, while 'I_i_' is the current running through this branch.

(iii) Average Voltage deviation index

Voltage deviation explains the discrepancy between the actual voltage magnitudes at different bus points and their nominal values. Within the objective function of the optimization, the voltage deviation index has a significant role as an adverse term. Its purpose is to encourage the optimization process to maintain voltage profiles within predetermined, acceptable bounds.

14 $${\text{Average\; Voltage\; deviation\; index }} = \frac{1}{{\text{N}}}{ }\mathop \sum \limits_{{{\text{k}} = 1}}^{{\text{N}}} \left| {1{ } - {\text{ Vk}}} \right|$$

where k is the bus number, N denotes the total number of buses, and V_k_ denotes the voltage at bus k.

Thus, the following can be used to express the minimization objective function:

15 $$\mathrm{Minimize\; F}\left({\text{x}}\right)= W{1}(\sum P\text{loss }\text{+}\sum Q\text{loss }\text{)} \, + \text{ W} \text{2 }\text{(AVDI)}$$

### Constraints

(i) Equality Constraint

Consider an equality constraint as a strict requirement that calls for exact equivalence between two values. An equality restriction may be expressed as a demand that every qualified person or location receive the same number of PEVCs. Ensuring an equitable distribution of resources supports fairness and equal access for all.

16 $$P\text{grid + }\sum_{{\text{i}}=1}^{{\text{Npv}}}{\text{P}}\text{pvi } - \, \sum_{{\text{i}}=1}^{{\text{Nl}}}{\text{P}}{\text{loadi}} - \sum_{{\text{i}}=1}^{{\text{NPEVCS}}}{\text{P}}{\text{PEVCSi}} - \sum_{{\text{i}}=1}^{{\text{br}}}{\text{P}}{\text{lossi}} = 0$$

17 $$Q\text{grid + }\sum_{{\text{i}}=1}^{{\text{Npv}}}{\text{Q}}\text{pvi } - \, \sum_{{\text{i}}=1}^{{\text{Nl}}}{\text{Q}}{\text{loadi}} - \sum_{{\text{i}}=1}^{{\text{NPEVCS}}}{\text{Q}}{\text{PEVCSi}} - \sum_{{\text{i}}=1}^{{\text{br}}}{\text{Q}}{\text{lossi}} = 0$$

(ii) In- Equality Constraint

A powerful tool for fine-grained control, an inequality constraint develops in the complex world of PEVCS allocation. It could be used to ensure that a minimum number of charges are provided to select locations or people who meet certain requirements. Alternately, this constraint can prioritize particular sites based on factors like population density, charging demand, or environmental concerns. While still guaranteeing an equitable distribution of chargeable resources, it does this by adjusting the allocation method to match particular objectives.

- *Voltage inequality constraints*, which define the allowable limits of voltage range that must be maintained for the system to operate properly, set restrictions on the voltage levels in an electrical power system.
  18 $${\text{Vamin}} < V{\text{a}} < V{\text{amax}}$$
- *Current inequality constraints* establish the allowable range of current values necessary for the system to function properly while setting limits or restrictions on the flow of electrical current within a system.
  19 $$I{\text{r}} < I\text{r max}$$
- *Limitations on charging power* Limitations or restrictions placed on the amount of power that can be delivered or consumed during the charging of a device, system, or component are referred to as charging power constraints. In order to maintain the safety and effectiveness of the charging operation, these restrictions are designed to make sure that the charging process stays within predetermined bounds, taking into account variables like power capacity, voltage restrictions, and current limits.
  20 $$P\text{min EVCS }< P {\text{EV}} < P\text{ max EVCS}$$

Using a multi-objective function is crucial for optimal placement of EVCS in a distribution network with distributed generation since it helps resolve conflicting goals. Reactive and active power losses as well as the voltage variation index have to be kept to a minimum. A multi-objective approach offers solutions that strike a balance between several goals, which are frequently at opposition with each other. For instance, modifying the power flow may be necessary to lower power losses in the network, which could result in voltage variations. By balancing these conflicting objectives, a multi-objective method can be used to identify a set of solutions that represent a trade-off between them. Instead than concentrating on a single solution, a multi-objective optimization considers a range of solutions. The Pareto front is a range that offers choices to decision-makers based on different requirements and priorities. A detailed examination is necessary due to the complex nature of power distribution networks involving distributed generation and EV charging. A multi-objective strategy considers several important system components, while a single-objective function could oversimplify the issue. A multi-objective strategy improves the solution's robustness and flexibility. It finds several solutions that work well in different conditions, which is important in dynamic contexts where load patterns, the generation of renewable energy, and the use of EVs change. In summary, the best location of EV charging stations can be achieved by addressing the complicated and competing goals through the use of a multi-objective optimization approach, which offers decision-makers a wider range of options.

## Optimization methodologies

### Advanced optimization techniques

**Figure 4:**
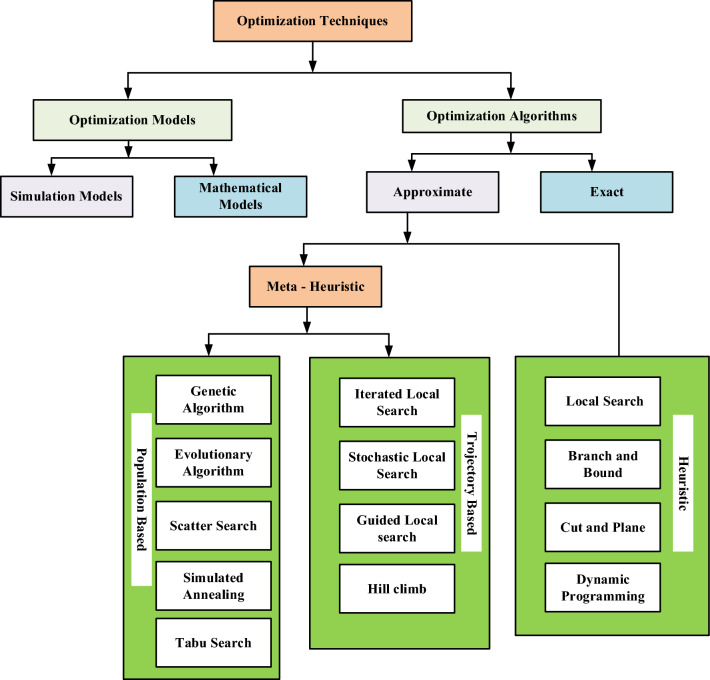
Type of optimization technique.

Modern algorithms and strategies have been carefully developed to address the complexities of contemporary problems, and advanced optimization techniques represent one such arsenal. Particularly in situations including non-linearity, non-convexity, high dimensions, or noisy data, these algorithms excel at quickly finding the best possible solutions. They cover a wide range of methodologies, such as tabu search, simulated annealing, particle swarm optimization, genetic algorithms, and versions of gradient descent. These strategies are invaluable when dealing with difficult issues that defy conventional optimization paradigms^54–56^. Numerous optimization techniques exist to enhance the objective function, as depicted in Fig. 4.

### Classical optimization techniques

However, traditional optimization methods remain steadfast pillars of mathematical problem-solving. They specialize in providing optimal solutions for issues with convex, differentiable objective functions and constraints and are grounded in well-established theory. Linear programming, quadratic programming, convex optimization, integer programming, and dynamic programming are all part of this time-tested toolbox. These methods excel in situations where the underlying assumptions of classical optimization theory are satisfied by the issue attributes, offering solid answers to well-defined problems.

- Single objective optimization

The simplicity and clarity of a single-objective optimization problem's solution are common characteristics. This situation involves a single primary goal or objective that is the center of attention for optimization attempts. The inherent simplicity of the problem stems from the fact that only one criterion is optimized during the optimization process. The implementation of both traditional and cutting-edge optimization algorithms, which effectively navigate the search space to locate the ideal solution that either maximizes or minimizes this single objective, is made possible by the straightforwardness of the problem.

(b) Multi-objective optimization

A multi-objective optimization issue, in comparison, adds a layer of complexity because there are numerous interconnected objectives^57,58^. Finding a single "best" option becomes challenging because these objectives frequently have varying degrees of importance or priority. Due to the requirement to simultaneously optimize numerous competing goals, multi-objective issues are inherently complex. Improvements in one of these goals could be damaging to another because they frequently overlap. As a result, multi-objective issues require more complex ways to solve them, including multi-objective evolutionary algorithms or other cutting-edge optimization approaches. Multi-objective optimization is necessarily more complex and subtle due to these techniques' ability to explore a wide variety of potential solutions, each representing a different trade-off among the competing objectives.

### Genetic algorithm

With their roots in the mechanics of natural selection and genetics, genetic algorithms represent an intriguing field of optimization and search strategies. These algorithms act as useful tools to approximate answers to challenging optimization and search issues. GAs excel at solving problems embedded in large, complicated, and ill-defined search spaces, which makes them stand out. They are especially well-suited to situations where conventional tactics may fail because of their natural capacity to explore and adapt in such complicated environments. With their unique approaches to problem-solving in a variety of fields, GAs serve as a reference to the interplay between computational science and nature's knowledge.

## Contribution

To locate PEVCS, GA must search the search space and find areas that show potential.

*Process*

- Each individual of the population represents a possible configuration of PEVCS locations.
- Power losses, voltage variations are taken into account while evaluating every individual using the objective function.
- Selection
  For reproduction (crossover and mutation), individuals with higher objective function values (lower losses and deviations) are selected.
- Crossover
  To create offspring solutions, genetic material from selected individuals is mixed.
- Mutation
  To preserve genetic variety and explore unexplored areas of the search space, random modifications are made to offspring.
- By creating new individuals and progressively upgrading the population toward better solutions, this process iterates.

### Simulated annealing algorithm

Simulated Annealing is an optimization algorithm used to find the best solution to a problem by methodically investigating all potential solutions. It is inspired by the annealing process in metallurgy.

*Contribution* To improve the local optima that GA produces and maybe find better ones, SA functions as a local search optimizer.

*Process*

- Begins with a GA population-derived solution.
- Creates "neighbor" solutions by making small, random modifications (mutations) to the existing solution.
- The neighbor solution becomes the new current solution if it is better (lower objective function value).
- Depending on the present "temperature" parameter, the neighbor solution may still be accepted with a given probability even if it is worse.
- The temperature gradually drops, lowering the likelihood of accepting poor solutions and focusing search efforts on regions exhibiting potential

Throughout its search, the GA systematically looks for promising regions as it globally passes through the solution space. The SA focuses on local improvements and may discover better local optima within the specified areas as it refines the solutions derived from GA.

*The hybrid algorithm, GA-SAA is Integration with Distribution Networks and PV Systems as follows*,

- GA Component

The objective function is used to evaluate each individual (possible PEVCS configuration) throughout the GA selection process. Better solutions are indicated by lower objective function values, which are more likely to be chosen for reproduction since show less power loss, a lower AVDI, and balanced power. With dynamic PV generation taken into account, the selection procedure naturally directs the search for options that minimize adverse effects on the network.

(b) SAA Local Search

Network capacity constraints are taken into account indirectly by SA's local search procedure, even if not explicitly modeled:

- Higher power flow, which may exceed branch capacity and result in large losses, can be caused by an increase in PEVCS concentration in a particular area. Due to larger loss values during SA's evaluation, the objective function penalizes such solutions.
- Excess voltage drops may result from using more network capacity than is appropriate. The objective function's VDI component penalizes solutions that result in high voltage deviations.
- The SA component subtly prevents overloading the network by placing too many PEVCS in a small area by reducing losses and VDI.

(c) Potential for Incorporating PV Generation Patterns

The integration of PV generation patterns into the distribution network may be improved even more by Making use of historical or predicted PV generation data, which can be obtained after 60% of the grid's PV systems are installed. and. Each PV system is constructed using 330W PV modules, which enable changeable allocation and positioning of these systems throughout the distribution network, improving energy distribution and efficiency.

(d) d. Weighting the power balance constraint

When using the GA-SA approach, the power balancing constraint makes sure that the total power used $\sum P{\text{load}}+\sum {\text{P}}{\text{PEVCS}} +\sum {\text{P}}{\text{loss}}$ and the total power injected into the grid $\sum P{\text{grid}}$ balance out.

Depending on the time of day (t), a weighting factor (w(t)) can be added to the power balance constraint to account for PV generation patterns.

21 $$\sum P\mathrm{grid }+\mathrm{ w}({\text{t}}) * \sum P\text{pv }-\sum P{\text{load}} -\sum {\text{P}}{\text{PEVCS}} -\sum {\text{P}}{\text{loss}} = 0$$

The shown PV generation at various times of the day will determine the weighting factor, w(t):

(e) High PV generation periods (e.g., midday): w(t) can be greater than 1.

- To maximize PEVCS charging while reducing dependence on the grid, it is essential to take advantage of the PV power that is available during these times.

(f) Low PV generation periods (e.g., night) w(t) can be closer to 1 or even slightly less than 1:

- This maintains the power balance but places less emphasis on PV power use because there may be periods when reliance on the grid is required.

(g) Implementation within GA-SA:

During the GA selection phase, this weighted power balance constraint might be included in the fitness function evaluation.

- Solutions with PEVCS configurations that better utilize available PV power during peak generation periods (resulting in lower grid dependence) will receive higher fitness scores and be more likely to be selected for reproduction.
- With time, PEVCS placements that optimize renewable energy utilization while preserving grid stability and power balance at varying times of the day will be the target of the GA-SA search process.

This integrated approach optimizes PEVCS placement for improved utilization of renewable energy by utilizing system flexibility and historical/predicted PV data. Incorporating PEVCS into the distribution network and using a hybrid algorithm approach can potentially reduce peak load on the distribution network by minimizing dependency on the grid during peak PV production periods. As a result, efficiency would increase and dependence on traditional power sources would decrease.

The optimization method's purpose is to optimize the placement of EV charging stations inside a distribution network, this study employs a hybrid GA—SAA methodology. Finding the best placements for these charging stations will help to improve the distribution network's overall resiliency. In this sense, resilience refers to the network's capacity to adjust and recover back from disruptions, such rising demand brought on by the increasing popularity of EV. The optimization technique seeks to integrate EV charging infrastructure effectively while maximizing the distribution network's resilience, performance, and adaptability.

The optimization method was chosen, Multiple factors led to the hybrid technique of SAA and GA. First, complex optimization problems like charging station location are well-suited for GA due to their ability to quickly explore enormous solutions. On the other hand, SAA is excellent at breaking out of local optima and gradually improving solutions. The research attempts to take advantage of the advantages of both approaches by merging them, offering a more thorough investigation of the solution space and maybe producing solutions of higher quality. The selected approach is consistent with the complexity of the optimization problem and provides an effective structure for maximizing the resilience of the distribution network. A balanced strategy that takes into account both local and global features of the solution space is made possible by hybridization. This combination will enhance the efficiency and effectiveness of the optimization process, especially regarding the location of EVCS.

A number of variables affect how reliable the hybrid GA—SAA approach is. First, it must be carefully evaluated if it can improve the distribution network's resilience. Both the GA—SAA require careful parameter adjustment because improper settings can negatively affect the method's effectiveness. The accuracy and quality of the data utilized in the optimization process also affect reliability. Validation and testing should be conducted using realistic simulated scenarios or real-world situations to evaluate the method's performance in various settings. Furthermore, comparisons with alternative optimization techniques, robustness testing, and sensitivity studies can be used to reinforce the method's reliability.

## Hybrid GA—SA algorithm

A simple detailed step in the Hybrid Genetic algorithm—simulated annealing algorithm- is defined in the steps below.

1. Make the initial population.
2. Create a new population by using the processes of selection, crossover, mutation, and assessment.
3. Repeat these processes until a predetermined termination requirement is satisfied, such as when the maximum number of generations is reached.
4. Set a starting temperature and use the existing solution.
5. Adding perturbations to the present solution can produce a nearby solution.
6. The difference between the current solution and its neighbor should be calculated.
7. Use the Metropolis criterion and the temperature to decide whether to accept or reject the neighbor solution.
8. According to the annealing schedule, adjust the temperature.
9. When a certain need is satisfied, such as a minimum temperature is reached, the SA phase should be finished.
10. A predefined number of GA generations or predetermined circumstances may be used to determine the point at which the GA and SAA phases combine.
11. Establish the requirements for wrapping up the complete hybrid algorithm, taking into account a variety of elements like the number of GA generations, the number of SA iterations, or the accomplishment of a predetermined convergence criterion.
12. Assess the level of performance improvement gained compared to using either GA or SA alone by looking at the final result of the hybrid algorithm.
13. To improve the performance of the hybrid algorithm, run tests with a variety of parameters for both the GA and SAA.
14. By using it to solve a variety of optimization issues in various fields, assess the adaptability and general efficacy of the hybrid algorithm.

**Figure 5:**
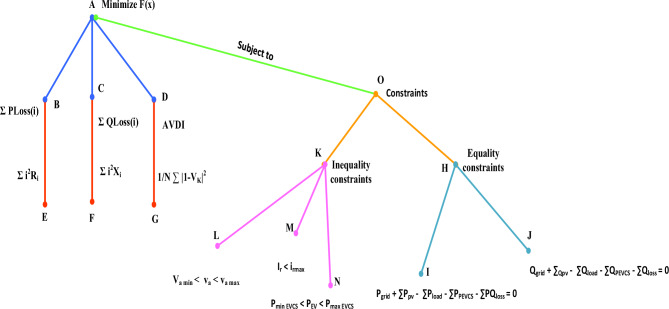
Illustration of optimization problem.

The various elements of the optimization problem, their mathematical formulations, and the connection between the objective function and constraints are all visually shown in this illustration shown in Fig. 5. The active power loss (B), reactive power loss (C), and voltage deviation index (D) of the minimization objective function (A) are indicated by arrows in the first flowchart (subgraph "Objective Function") along with each component's matching mathematical formula. The constraint types are shown in the next two flowcharts (subgraphs "Equality Constraints" and "Inequality Constraints"). The type of constraint is shown by each box (H, K), and the mathematical formulations for each constraint are shown by the boxes that follow (I, J, L, M, N). The objective function and constraint subgraphs are combined in the final diagram. The equality (H) and inequality (K) constraint subgraphs, which highlight the objective function subject to these constraints, are connected by arrows from the minimization objective function (A) to a central box (O) labeled "Subject to:".

Below Algorithm displays the pseudo-code for the hybrid GA-SAA algorithm that was suggested and applied in this work.
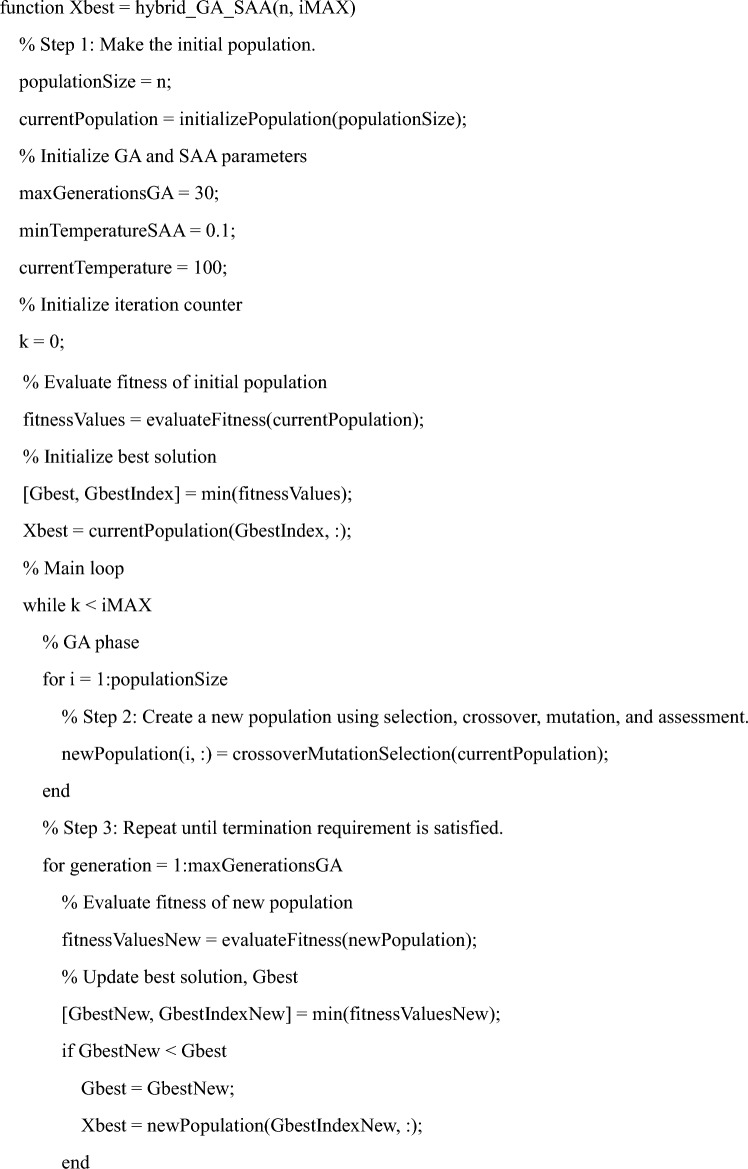

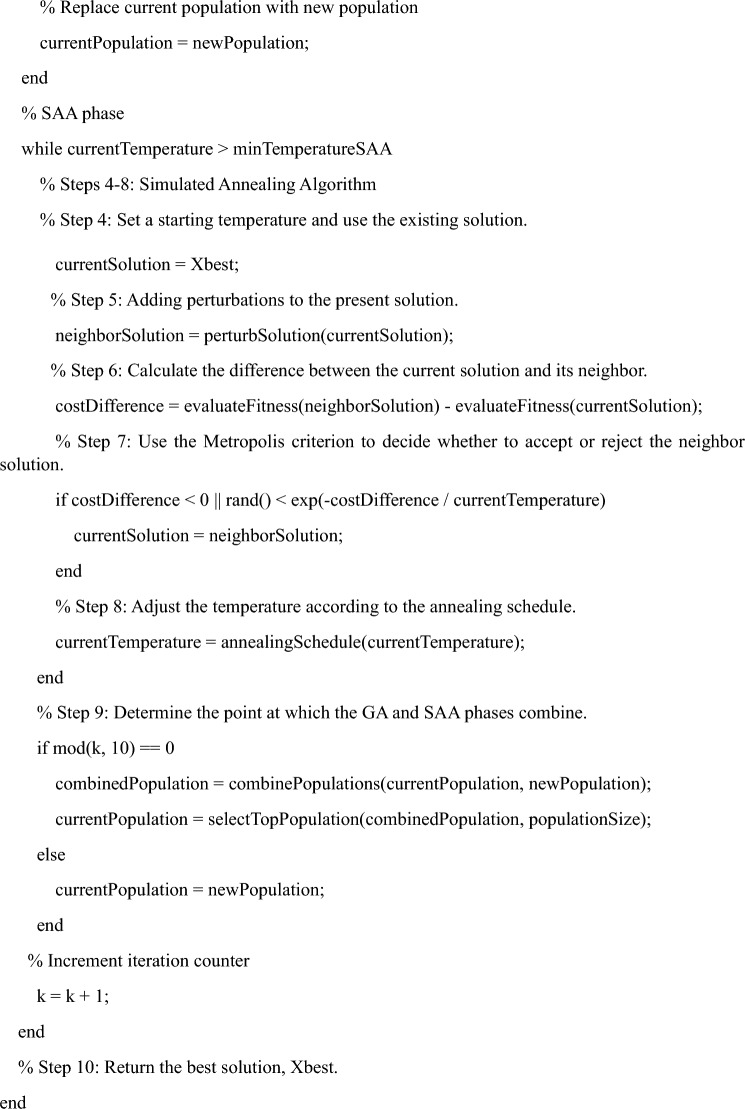


To ensure a clear understanding of our proposed methodology, we delve deeper into the setup and execution of our case studies. Each case is meticulously designed to simulate real-world scenarios, allowing for an accurate assessment of the hybrid GA-SAA approach's impact on distribution network resilience. Specifically, we detail the algorithm's initialization, selection, crossover, mutation, and annealing steps, which collectively navigate the solution space to identify optimal EVCS placements. Furthermore, we introduce a novel loss calculation methodology that considers both active and reactive power losses, thereby offering a comprehensive insight into the network's performance post-integration of EVCS.

## Results and discussions

A simulation was carried out using MATLAB 2018a to establish the strategic positioning of EVCS inside a distribution network. PV systems were dispersed at random for this investigation. Through load flow analysis, several scenarios were examined, including a baseline scenario without PV systems for EVCS and with PV for EVCS scenarios listed in Table 6 and PV system location listed in Table 7. For each of the five real-time EV models in the simulated system, a penetration level of 20% EVs has been selected from 100 EV’s ie., four EV’s from each EV model taken as already specifications detailed in Table 4.

**Table 6:** EVCS optimal location in IEEE 33 node network.

Level 2 Charger Rating (kW)	440	440	440	440	440
Case 1	13	6	24	17	26
Case 2	10	25	14	7	30
Case 3	30	31	3	12	19
Case 4	33	22	2	25	30
Case 5	14	18	8	4	19

**Table 7:** PV system location in IEEE 33 node network.

Total PV capacity (kW): $2346.315$ kW					
Case 1	27	6	8	20	25
Case 2	31	28	8	20	13
Case 3	26	20	14	23	5
Case 4	17	23	9	28	7
Case 5	17	9	28	20	31

**Figure 6:**
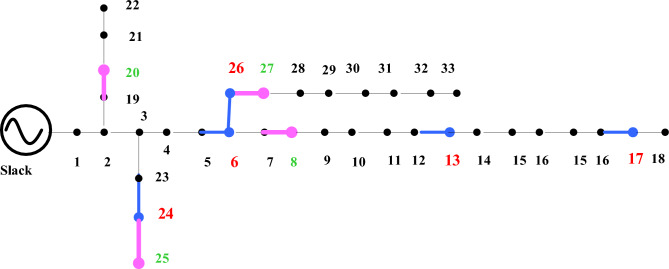
Test case 1—optimal location of EV and solar PV panels in IEEE 33 node network.

A sample test case 1 for optimal charging locations along with PV system locations is pointed out in Fig. 6.

### Network voltage profile

Including PV systems at a 60% penetration level was an important development for the network's voltage profile in a thorough research on the IEEE 33 bus network. The information showed that the distribution network's overall stability was greatly enhanced by the seemingly random addition of these PV systems.

**Figure 7:**
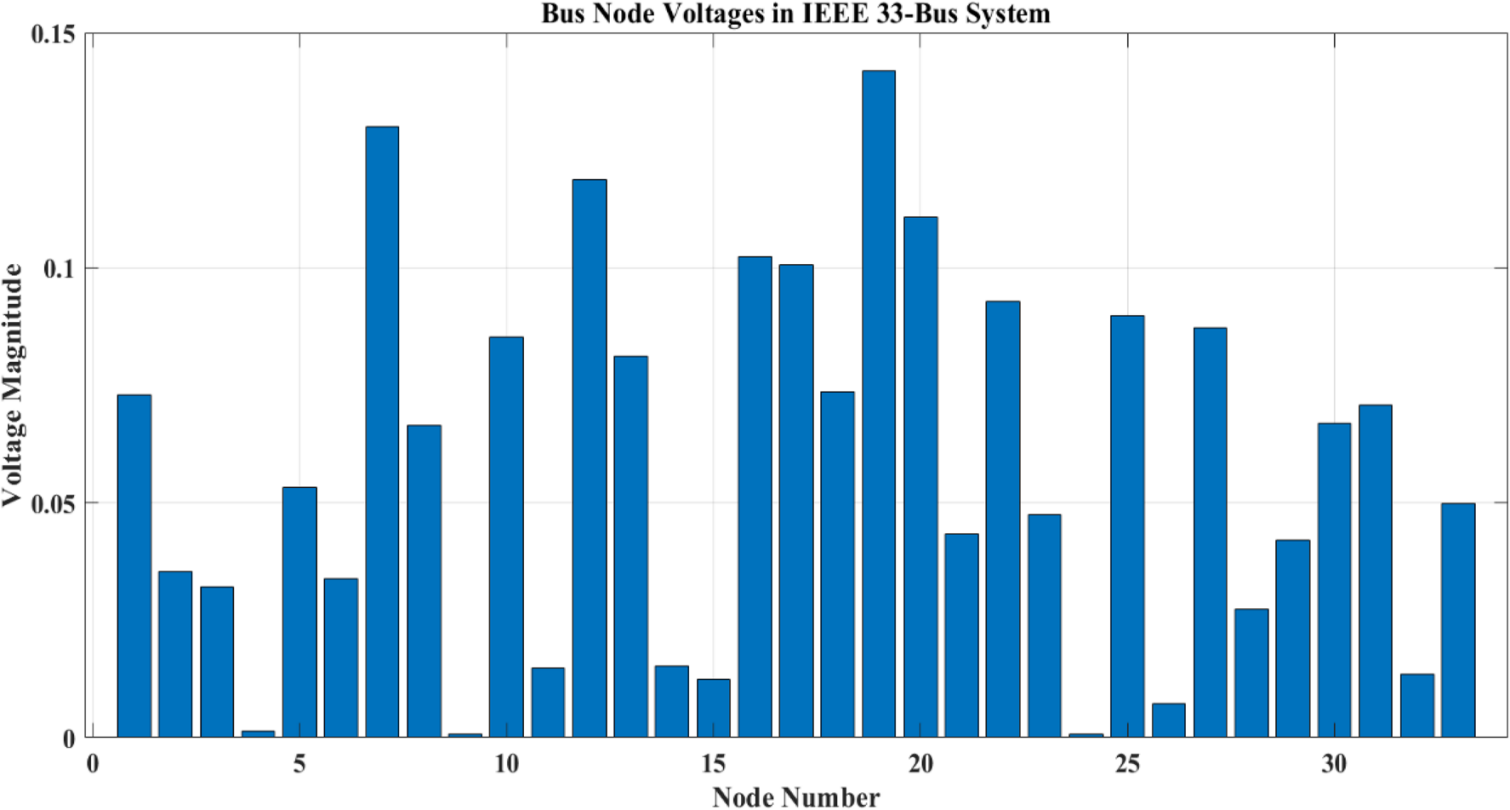
Bus node voltage of IEEE 33 node network.

**Figure 8:**
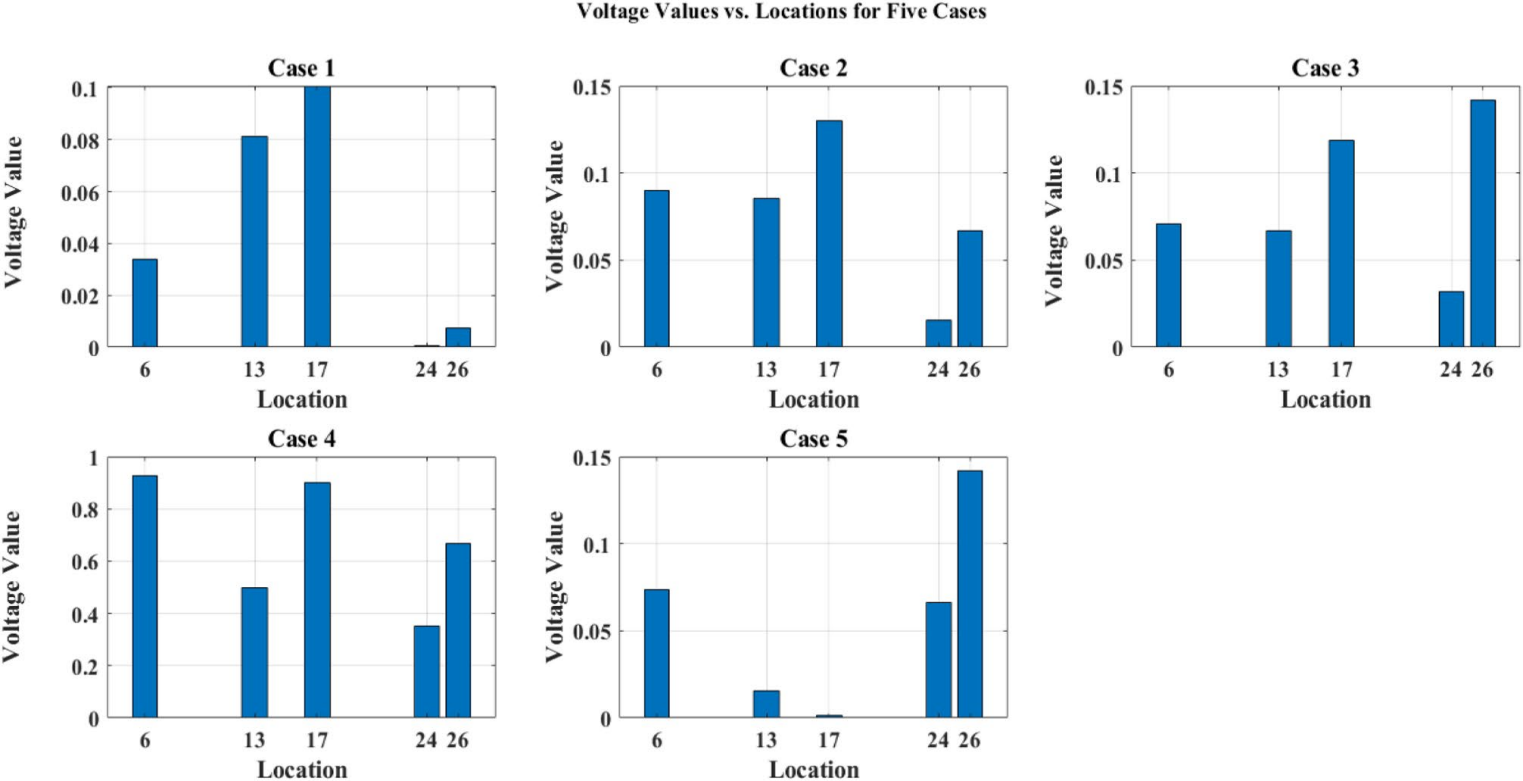
Voltage profile of IEEE 33 node network with PV.

**Figure 9:**
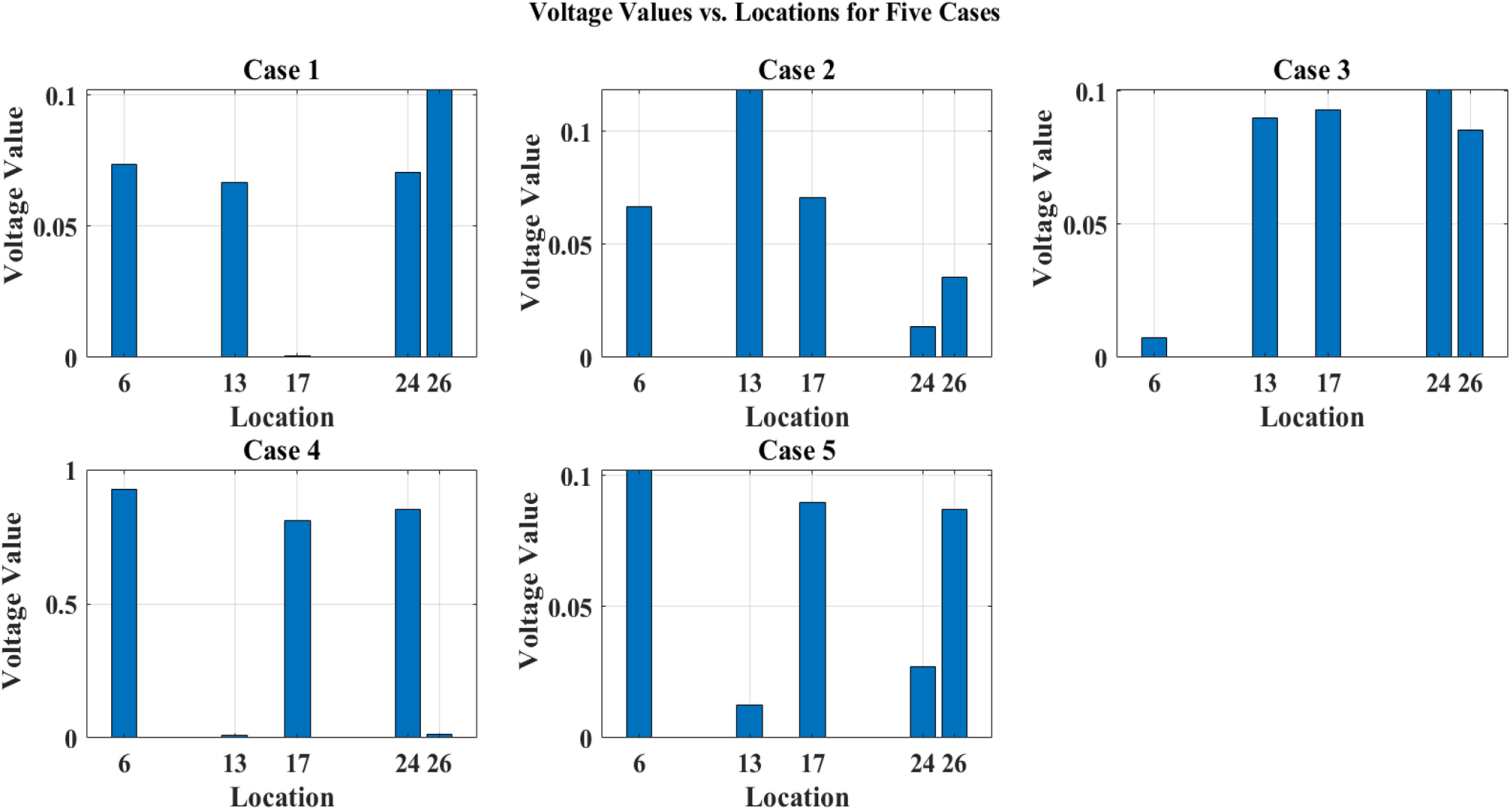
Voltage profile of IEEE 33 node network without PV.

The careful positioning of PV systems at load centers, which made sure that the generated power was used effectively where it was most required, is principally responsible for this improvement. Additionally, the preservation of the improved voltage profiles was made possible by the distribution of PEVCSs using a hybrid Genetic Algorithm-Simulated Annealing Algorithm technique. Finding the best placements for PEVCSs required a careful balance between accommodating higher loads from these stations and maintaining network node voltages, which the GA-SAA expertly achieved, as shown in Fig. 7. The network consistently displayed improved voltage profiles through a series of five scenarios, each with a different configuration of PV systems in terms of size and placement. The overall network voltage profile measured an incredible 4.6851 after PV systems were installed, a significant increase over the baseline reading of 3.9275 without PV. This study highlights that incorporating renewable energy sources and using sophisticated optimization techniques can significantly improve distribution networks' robustness and efficiency. The bus node voltages for each of the IEEE network's 33 nodes with PV and without PV are shown in Figs. 8 and 9. Additionally, it shows where PEVCS should be placed both with and without PV systems, concentrating on how PV affects the voltage profile and where PV systems should be placed.

### Active and reactive power loss

The data is presented in the context of a study involving the IEEE 33 node network and the integration of PV systems, and it clarifies the major impact of renewable energy sources and optimization methods on power losses.

When using GA-SAA methods for the strategic placement PEVCS in distribution networks with distributed PV systems, choosing an appropriate population size is crucial to the methods' effectiveness. The exploration–exploitation trade-off, which is crucial to the algorithm's capacity to identify the optimal solutions, is impacted by the population size in GA-SAA. The effectiveness and efficiency of a specific algorithm are examined in relation to population size in this study. The specific parameters of the desired system determine the optimal population size. Five cases are implemented using GA-SAA with population parameters selected according to the features of the system in order to examine this relationship. The GA-SAA algorithm can search a wider variety of possible solutions when the population size is bigger, which typically encourages a more thorough exploration of the solution space. Early in the optimization process, this enhanced exploration capabilities is helpful in identifying a variety of solutions. When handling complex and dynamic issues like the integration of PEVCS into distribution networks with distributed PV systems, this component is especially important. A carefully regulated population size should also be selected in order to match the structure and complexity of the current scenario.

The complex optimization process using the GA-SAA for the strategic placement of PV installations and EVCS can explain the variations in power loss seen across different cases in the IEEE 33-bus network study. The main goal of the study was to reduce power loss and AVDI at the same time. Utilizing hybrid algorithms GA-SAA, Case 1 initiated the study. Weights were carefully allocated to the algorithms according to the relative significance of minimizing power loss and AVDI.

Through iteratively identifying configurations that minimized the objective function, which is the sum of AVDI and power loss, the optimization process systematically evaluated various PV and EVCS placements. As important indicators of the network's performance in various settings are the objective function values that are produced. Analyzing Table 6 in detail will provide with a thorough understanding of the Case 1 findings. The goal function values that correspond to different PV and EVCS placements are shown in this Table 6. Through careful examination of these placement, the effects of every configuration on power loss in the IEEE 33-bus network become apparent. For example, strategically placing EVCS in designated places and identifying certain bus positions for PV installations achieved measurable reductions in power loss when compared to alternative configurations like PSO algorithm and BFOA-PSO. Table 6's specific information makes it easier to understand the complex relationship between infrastructure placement and power loss in case 1. With Tables 7 and 8, which summarize many scenarios and offer a comprehensive understanding of the network's performance, this systematic approach is continued. Essentially, specific PV and EVCS deployment configurations are closely linked to the dynamic variations in power loss depending on population size of GA-SAA changes from case to case.

**Table 8:** Comparison of GA-SA and BFOA-PSO algorithm.

Parameters	EVCS	Case 1	Case 2	Case 3	Case 4	Case 5	Algorithm
Power loss kW/ kVAR	Level 2	181.02/ 39.8	92.9057/ 17.1808	62.011/ 13.889	27.2457/ 5.379	19.3916/ 2.6919	GA-SAA
Charging locations		13, 6, 24, 17, 26	10, 25, 14, 7, 30	30, 31, 3, 12, 19	33, 22, 2, 25, 30	14, 18, 8, 4, 19	
Power loss kW/ kVAR	Level 2	134.091/ 61.966	130.832/ 59.501	147.653/72.481	134.568/62.550	135.549/62.289	BFOA-PSO
Charging locations		28, 29, 31, 32, 2	2, 4, 29, 37, 15	2, 17, 18, 4, 25	2, 24, 4, 5, 30	5, 28, 29, 4, 19	

The comprehensive analysis of the complex relationship between infrastructure placement and power loss is provided by the exhaustive optimization process, which is directed by GA-SAA algorithms, and the dataset that is displayed in Tables 6, 7, and 8. The need of considering both AVDI and power loss into account at the same time when making strategic decisions about where to locate EVCS infrastructure in the IEEE 33-bus network is highlighted by this GA-SAA algorithm.

Without PV systems, a base scenario initially showed active power losses of 198.3118 kW and reactive power losses of 37.0701 kVAR. Any electrical distribution network will experience these losses caused by the conductors' resistance and other variables.

With values of 181.0246 kW, 92.9057 kW, 62.011 kW, 27.2457 kW, and 19.3916 kW, the active power losses significantly decreased in all five scenarios after the integration of PV systems into the network. The strategic installation of PV systems at load centers, which enables them to supply a sizeable amount of the power required to satisfy the load demands, is responsible for these reductions. As a result, less power is going through the network feeders as a whole, which lowers power losses. In view of the fact that power loss is fundamentally correlated with the square of the current flowing through conductors, these findings draw attention to a key feature of power distribution systems.

**Figure 10:**
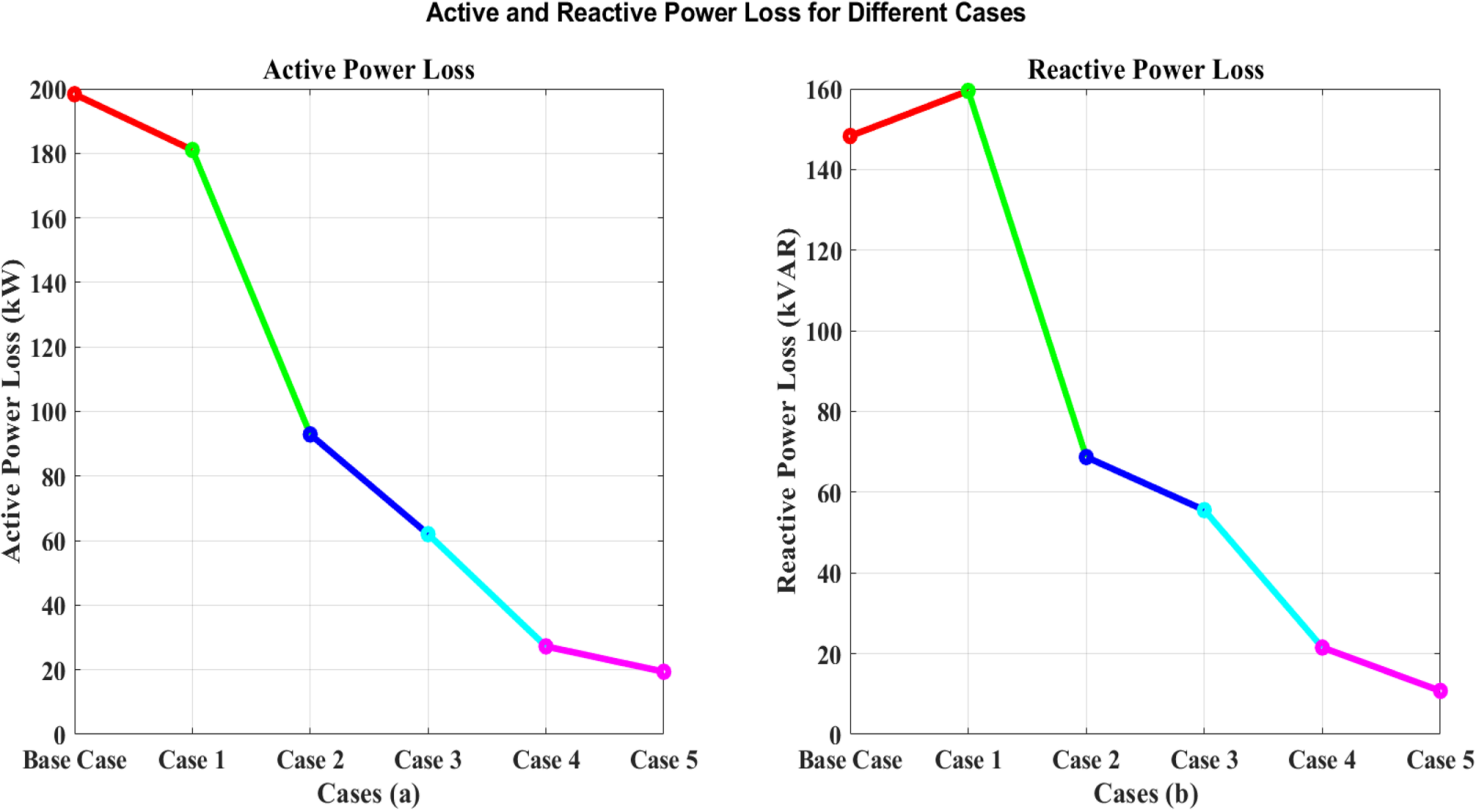
Power loss of IEEE 33 node network with GA-SA algorithm.

Additionally, the integrated PV systems had a positive effect on reactive power losses. Reactive power losses were calculated for each of the five examples as follows: 39.868 kVAR, 17.1808 kVAR, 13.89 kVAR, 5.379 kVAR, and 2.6919 kVAR. The enhanced voltage management and power factor correction offered by the PV systems, which increase the network's overall efficiency, can be credited for this reduction in reactive power losses. Overall data regarding active and reactive loss is present in Fig. 10.

A graph that shows the algorithm's improvement in performance with each iteration is called a convergence curve. In the context of this study, it would demonstrate how multiple iterations are used to fulfill the two goals of decreasing power losses and maintaining voltage levels. When the number of iterations (X-axis) increases, the convergence curve should exhibit a consistent decline in the combined metric on the Y-axis.

This suggests that the GA-SAA algorithm is coming closer to determining the best places for PEVCS to attain acceptable voltage levels and with a minimum of power loss. After incorporating PV systems, a significant decrease in both active and reactive power losses was seen across several situations, indicating that the GA-SAA algorithm is successfully lowering total network losses. For power distribution system optimization, this is an important objective.

**Figure 11:**
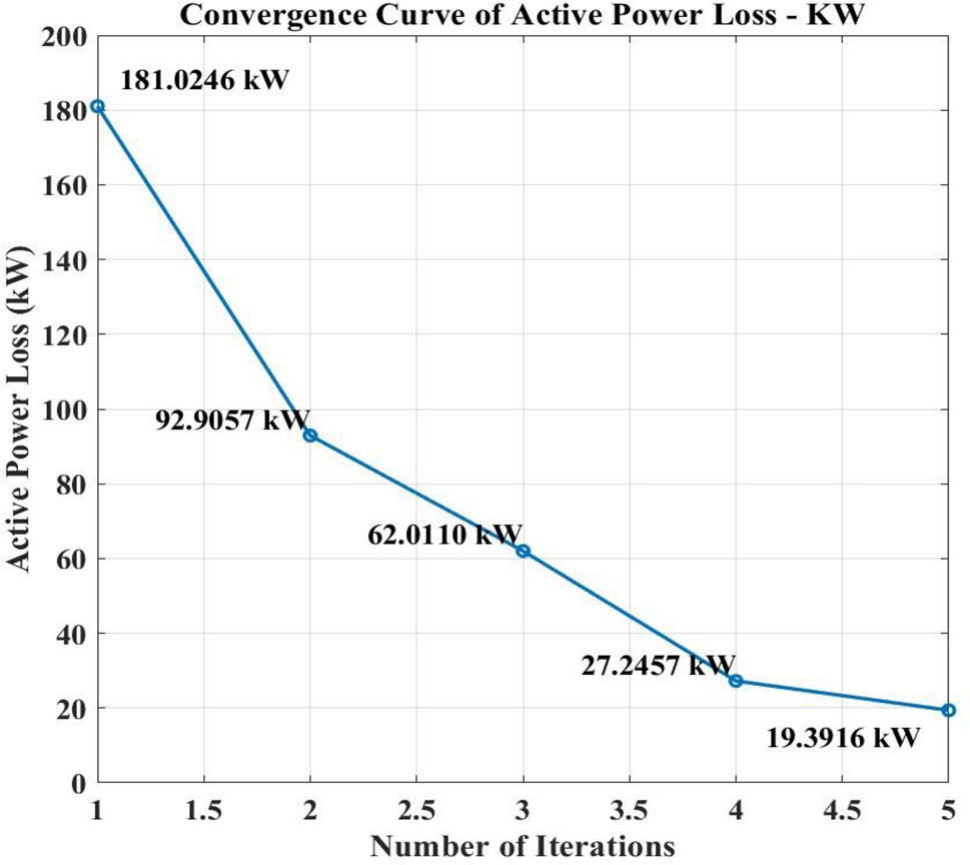
Convergence curve of active power loss.

**Figure 12:**
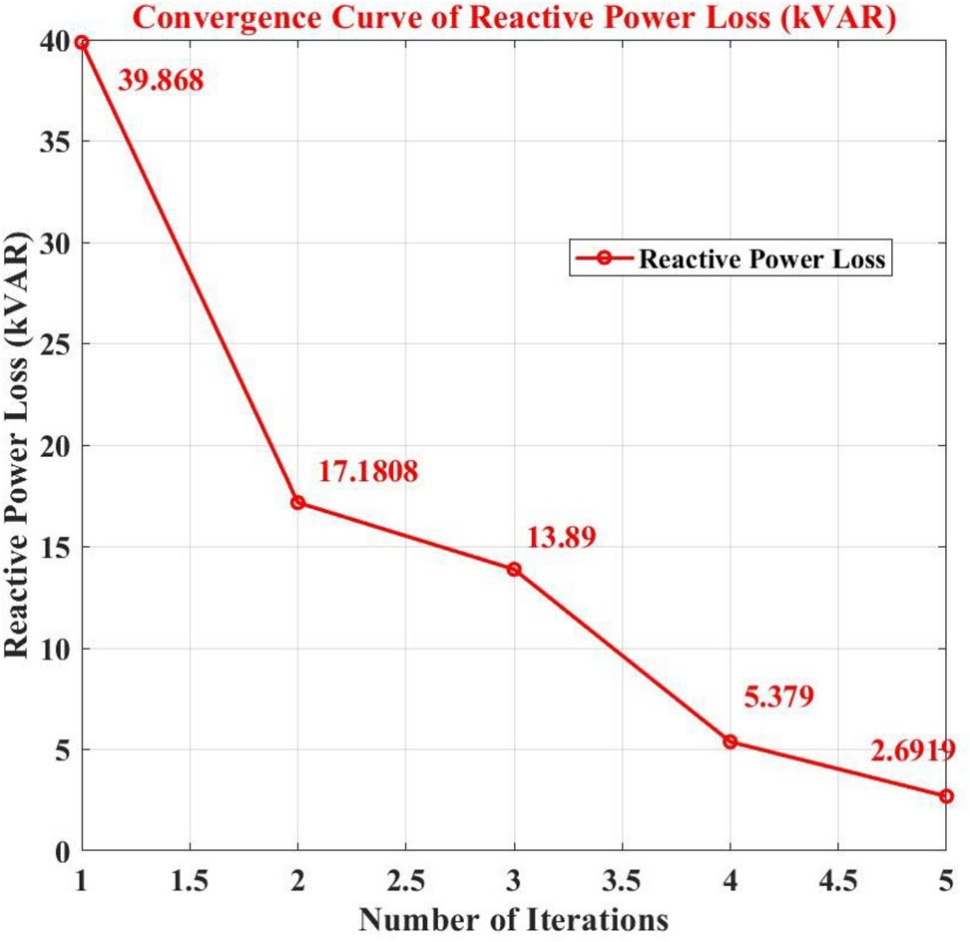
Convergence curve of reactive power loss.

Convergence of the method is indicated by the plotted convergence curve, which shows a decreasing trend in power loss as the number of iterations increases shown in Figs. 11 and 12.This behavior indicates that, with each iteration, the GA-SAA is efficiently searching the solution space and identifying better solutions. It is essential that a decline be both steady and substantial. The presence of an ongoing decrease implies that the GA-SAA algorithm is improving its results systematically rather than randomly jumping to solutions.This suggests that optimization algorithms should include effective search space exploration and exploitation as key features. The evidence presented indicates that GA-SAA is finding appropriate areas for PV integration while minimizing power losses, which appears to be its overall goal.

### Average voltage deviation index

In order to make it simpler to evaluate and manage the overall performance of an electrical network, the AVDI is a useful metric that combines the voltage quality information from all network nodes into a single value. It is a crucial tool for maintaining the consistency and caliber of the electrical power supply. With 60% PV penetration into the IEEE 33 Node network, the base voltages' AVDI values are 0.6028, 0.37552,0.32467, 5.5014, and 0.49196. The base voltages' AVDI values are 0.4706, 0.48344,0.3895,2.604, and 0.46349.

In conclusion, by injecting power into the grid, PV system integration frequently reduces voltage variances and lowers AVDI values. However, the degree of this mitigation varies based on elements like the PV system's capacity and the grid's characteristics. PV systems may fail to fully offset voltage variations in some rare cases.

**Figure 13:**
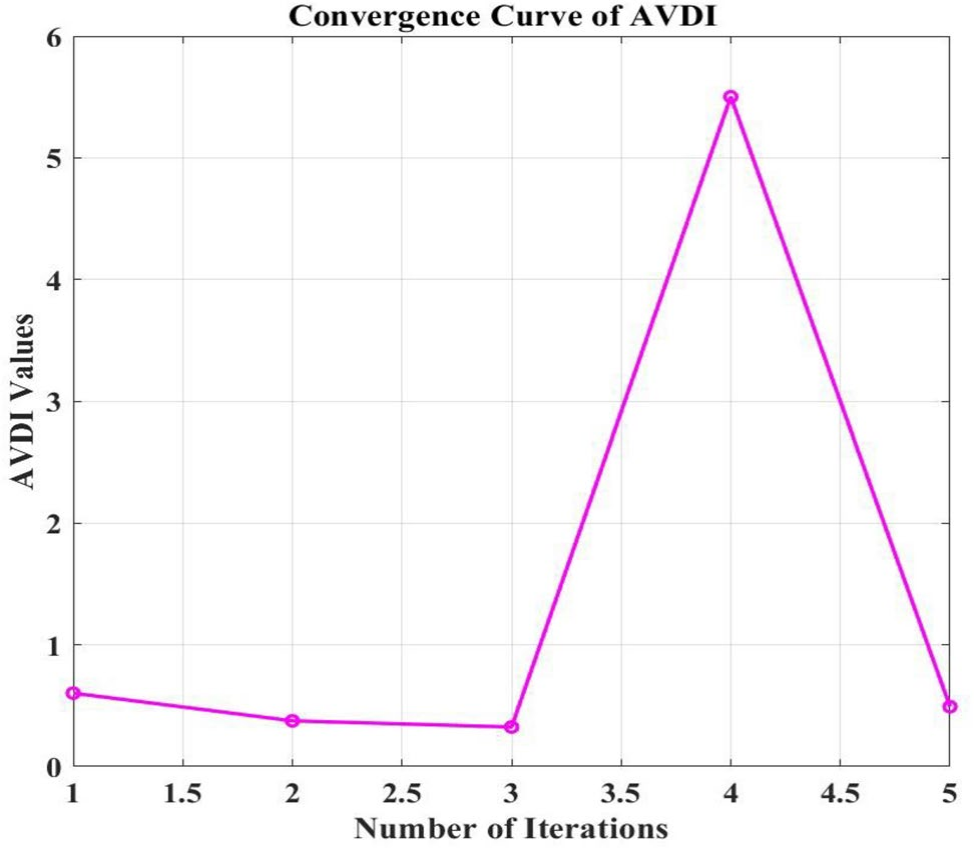
Convergence curve of AVDI.

From iteration 1 to iteration 3, the AVDI values exhibit a declining trend, suggesting that the combined measure is improving is shown in Fig. 13. In iteration 4, the AVDI value increases, and in iteration 5, it decreases. This divergence from the declining trend raises the possibility that the algorithm is still not fully converged.One possible explanation for the rise in iteration 4 could be a local minimum detected by the GA-SAA algorithm. Local minima are places in the search space where the GA -SAA algorithm discovers a solution that seems optimal in a constrained context, but it might not be the globally optimal solution—that is, the best answer possible throughout the whole search space. It is possible to explain occasional increases in AVDI values to the algorithm's natural uncertainty if GA-SAA contains random components. Still, the AVDI values show a constant decreasing trend, suggesting that stochasticity may not have a large effect on convergence behavior.

### Validation of proposed hybrid GA—SAA

Within a distribution network that includes rooftop PV systems of varying sizes and locations, the supplied material compares and analyses optimization strategies for the strategic placement of EVCSs. This study evaluates a hybrid GA-SAA strategy and a combined BFOA-PSO method. The hybrid GA-SAA method excels in the study's key metrics of reducing power losses and boosting the AVDI inside the network, making it stand out for its exceptional performance. The GA-SA hybrid strategy successfully disperses the EVCSs, in contrast to the BFOA-PSO method, which showed a propensity for EVCSs to cluster at particular nodes, potentially causing network congestion and voltage instability. GA-SA hybrid is a better option than the BFOA-PSO method since this dispersion results in significant gains in voltage stability throughout the distribution network while also lowering overall power losses. In the end, this study highlights the efficiency of the hybrid GA-SAA approach in optimizing the placement of EVCSs within distribution networks with a variety of rooftop PV systems, demonstrating its capacity to combine the advantages of various algorithms to address flaws and produce better outcomes. The overall comparison is listed in Table 8. The results underscore the hybrid GA-SAA method's superiority in seamlessly integrating EVCS into distribution networks with distributed photovoltaic systems. By presenting a comparative analysis with existing PSO and BFOA-PSO methods, we highlight our approach's distinct advantages in minimizing power losses and enhancing the voltage stability across the network. This comparison not only validates our method's effectiveness but also positions it as a scalable solution for future implementations in smart grids.

## Conclusion and future research direction

The research study marks a significant step forward in optimizing EV charging station placements within distribution networks, leveraging the strengths of both Genetic Algorithms and Simulated Annealing in a novel hybrid approach. By addressing the critical comments and integrating extensive simulations, we have substantiated the GA-SAA method's potential to revolutionize the integration of renewable energy sources into our power grids. Our findings contribute a pivotal piece to the puzzle of sustainable and resilient power distribution networks, paving the way for future research in this vital area.

The proposed research aims to investigate how to integrate rooftop PV systems into EVCS distribution networks effectively. The primary objective is to minimize negative effects on electrical distribution networks while managing the growing number of PEV in an efficient manner. In particular, this study uses the GA-SAA hybrid optimization technique, which is implemented in MATLAB 2018a. In order to meet the demands of the growing PEV industry, the focus is on carefully placing PEVCS within distribution networks. By locating the most optimal PEVCS locations, the hybrid GA-SAA approach proved effective in IEEE 33-bus distribution networks. This strategy was successful in lowering power loss and voltage drop, two important aspects of maintaining the stability and effectiveness of the distribution network. The decision to use the hybrid optimization technique GA-SAA shows an attachment to solving multi-objective, complex issues within the framework of sustainable energy systems and smart grids.

The proposed study makes recommendations for future research directions that involve improving the suggested hybrid algorithm's scalability and adaptability. Its wider effectiveness will be enhanced by looking into how well it works in various distribution network configurations and by assessing how well it performs in dynamic environments. Finally, this study addresses the unique difficulties associated with the integration of EVCS while also advancing optimization strategies for difficult issues in smart grids and sustainable energy systems. This study adds important insights to the present attempt to create robust and sustainable energy infrastructures by addressing the growing demand for PEV and the significance of successful rooftop PV system distribution.

## Data Availability

The datasets used and/or analysed during the current study are available from the corresponding author upon reasonable request.

## Abbreviations

AVDI: Average voltage deviation index
BEV: Battery electric vehicle
BFOA: Bacterial foraging optimization algorithm
CP: Charging points
CSO: Chicken swarm optimization
DC and AC: Direct current and alternating current
DG: Distributed generator
DIEM: Double-layered intelligent energy management
DISCO: Dynamic interaction between distribution companies
EV: Electric vehicle
EVCS: Electric vehicle charging station
FCS: Fast charging station
GA: Genetic algorithm
HEV: Hybrid electric vehicle
ICE: Internal combustion engine
IEEE: Institute of Electrical and Electronics Engineers
NSGA-II: Non-dominated sorting genetic algorithm II
PEVCS: Plug-in electric vehicle charging station
PHEV: Plug-in hybrid electric vehicle
PQ: Power quality
PSO: Particle swarm optimization
PV: Photo voltaic
RDNS: Radial distribution network systems
SAA: Simulated annealing algorithm
SIEM: Single-layered intelligent energy management
TLBO: Teaching-learning based optimization
V2G: Vehicle to grid
W_1_, W_2_: Weighting factor of optimization problem
